# Two-Dimensional Finite Element Analysis and Cutting Force Model for the Cutting of Circular Steel Bars Using Negative Rake Angle Cutters: Accounting for Chip Accumulation Effects

**DOI:** 10.3390/ma18061339

**Published:** 2025-03-18

**Authors:** Shifan Qiao, Chaobo Feng, Gang Wang, Taofu Liu, Jenisha Singh

**Affiliations:** 1 School of Civil Engineering, Central South University, Changsha 410075, China; qiaosf@csu.edu.cn (S.Q.); fengchaobo@csu.edu.cn (C.F.); 2 School of Advanced Interdisciplinary Studies, Hunan University of Technology and Business, Changsha 410205, China; 3 Industrialization Department, Xiangjiang Laboratory, Changsha 410205, China; 4 International Business School, Hunan University of Technology and Business, Changsha 410205, China

**Keywords:** shield tunnel cutting, cutting force calculation model, slip line theory, negative rake angle cutter, shear band mechanics, 2D finite element analysis, coupled Eulerian–Lagrangian (CEL) method, Johnson–Cook model, chip accumulation, orthogonal cutting

## Abstract

The cutting force exerted on steel bars plays a crucial role in determining tunneling parameters for shield tunneling, especially when cutters are used to cut through existing pile foundations. This research focuses on the cutting force during the initial phase of the cutting process. Using 2D finite element analysis, this study examines the early stage of orthogonal cutting with negative rake angle cutters, exploring the formation of a slip plane mode. By combining slip line theory with the shear band model, a computational model is developed to calculate the cutting force for negative rake angle cutters when cutting a circular steel bar cross-section at various depths. In addition, with the incorporation of the Johnson–Cook model, this study models cutting forces under various conditions, taking into account factors such as material strength, strain rate sensitivity, and temperature effects. The steels studied include AISI 1040, AISI 4340, and AISI 304, which are commonly used in construction, with attention given to how their mechanical properties, such as strength and hardness, affect the cutting forces. While this study acknowledges the steels’ manufacturing conditions, the primary focus remains on the cutting process and its impact on force predictions. The model’s calculated horizontal cutting force is compared to numerical simulations, showing a maximum absolute error of 33.85% and an average error of 14.23%. The vertical cutting force calculations are less accurate, with a maximum error of 64.2% and an average error of 14.06%. The analysis further reveals that chip accumulation significantly impacts the horizontal cutting force, while the variation in average stress along the slip line has a smaller effect. This study also examines how factors like material properties, initial temperature, low friction coefficients, and steel bar radius contribute to the model’s accuracy and reliability.

## 1. Introduction

With the continuous development of urbanization and the gradual increase in subway networks, the construction of shield tunnels in urban areas often involves dealing with the pile foundations of existing underground structures. With the development of shield technology and materials, shield machines have been developed to cut through piles directly by strengthening the shield cutter head and replacing the cutter. Currently, there are several cases where piles have been cut directly with the shield [1,2,3,4,5,6,7,8,9,10], for example, the Suzhou Guangji Bridge pile cutting project [2], the Beijing Metro Line 12 shield direct cutting of Xibahe Bridge pile project [7], and the Shenyang Metro Line 4 underpass Shenyang North Station pile cutting project [5]. However, the direct cutting of piles by shield is still far from being a cutting-edge technology.

Existing research on shield pile cutting mainly analyzes the thrust parameters of the entire cutter head and its impact on the surrounding environment based on experiments and actual engineering cases [9,11,12,13,14,15,16]. There are also some studies on cutting reinforced concrete [17,18,19,20,21,22,23,24,25,26,27,28,29], which mainly use numerical and experimental methods to analyze the cutting force of the entire cutter head or a single cutter cutting steel or concrete. When cutting the pile, the shield machine cuts the reinforced concrete material through the cutter on the cutter head. Different thrust parameters correspond to different cutting conditions, such as cutting speed and cutting depth. Different cutting configurations and parameters generate different cutting forces, which affect the cutter head torque and thrust of the shield machine. Therefore, a novel explanation of the cutting mechanism of the cutter when cutting reinforced concrete and accurately evaluating the cutting force are crucial for optimizing the thrust parameters and selecting the pile cutter. The selection of a suitable pile cutter and optimization of control parameters are crucial for energy conservation during tunnel construction, which aids the sustainable construction goal for any project [30,31,32].

Currently, when evaluating the cutting force of shield cutters, the finite element method is usually used to calculate the cutting force [33]. However, due to the characteristics of the finite element method itself and the complexity of the damage, considering the plastic constitutive model (Johnson–Cook model) used in the analysis of the cutting process, it is time-consuming to calculate the cutting force under different material parameters, cutter parameters, cutting depths, and other conditions.

Conversely, numerous studies have explored methods for calculating cutting forces in metals [34,35,36,37,38,39,40,41], including approaches such as slip line theory and the shear band model. These existing models are primarily designed to estimate cutting forces under steady-state cutting conditions in metal processing [37,38]. However, the process of cutting steel bars with shield cutters differs significantly from steady-state cutting. The duration of cutting from the start to the end is much shorter, and the boundary conditions are distinct from those in steady-state cutting. Furthermore, in steady-state and orthogonal cutting, the tool interacts with the workpiece as if it were cutting an infinitely long material. In contrast, when a shield cutter cuts a circular cross-section steel bar, it removes the upper circular segment above the cutting depth while leaving the lower segment intact due to its constraints. This results in boundary conditions that deviate from those of steady-state cutting. Consequently, existing cutting models are not well suited for calculating the cutting force of negative rake angle cutters when applied to circular cross-section steel bars.

One critical factor in accurately simulating the cutting process is the material’s yield strength and mechanical strength. A low-carbon steel commonly used in structural applications has lower yield strength and mechanical strength compared to higher-strength steels like AISI 4340, which is alloyed with nickel, chromium, and molybdenum. These differences in strength affect how the materials behave during cutting. Moreover, the influence of temperature on cutting forces, especially in high-speed cutting, cannot be overlooked. Temperature rise can soften the material, reducing cutting forces, whereas steel, which has lower strength, is more sensitive to temperature effects, particularly under high-speed cutting conditions. However, there remain significant gaps in the knowledge regarding the precise impact of high-speed cutting on the strength reduction of materials under elevated temperatures. This study aims to address these gaps by simulating cutting forces for various materials, considering not only the material properties but also the cutting parameters (e.g., rake angle, cutting depth) that influence the cutting process. The primary objectives are to develop a cutting force model that takes into account strain rate effects, temperature dependence, and material-specific properties, with the goal of improving cutting efficiency and tool lifespan in real-world applications.

This study focuses on the simulation of cutting forces during the initial phase of cutting steel rebars, specifically examining AISI 1040, AISI 4340, and AISI 304 steels. The cutting process is modeled using a 2D finite element analysis that incorporates the Johnson–Cook model to predict the material’s flow stress during high-strain-rate deformation. The proposed model accounts for strain, strain rate, and temperature effects, all of which are crucial in accurately simulating the cutting behavior of these materials. While the current study primarily focuses on the cutting process and cutting force predictions for different steel grades, it acknowledges that material properties such as microstructure, hardness, and mechanical strength play a vital role in determining cutting behavior. These material-specific properties are incorporated into the Johnson–Cook model, allowing for more accurate predictions of cutting forces under varying conditions.

In this paper, based on the numerical simulation results of the first cutting of two-dimensional circular cross-sections with negative rake angle cutters, based on the existing slip line theory and shear band model, a model for calculating the maximum cutting force suitable for the first cutting is proposed to reduce the time required for the preliminary estimation of horizontal cutting force. Considering different material parameters, the influence of initial temperature, the small friction coefficient condition, and the cross-section size of the steel bar, the results of the model calculation are compared with the numerical simulation results.

## 2. Numerical Analysis

The coupled Eulerian–Lagrangian (CEL) method of the Abaqus numerical simulation software (2021 version, Dassault Systèmes Simulia Corp., Vélizy-Villacoublay, France) is used to simulate the orthogonal cutting of the cutter and evaluate the cutting process of circular cross-section steel bars under different negative rake angles, cutting depths, and the change pattern of the slip plane (see Appendix A for detail). The slip face change model is analyzed.

### 2.1. Numerical Model Settings

**Figure 1:**
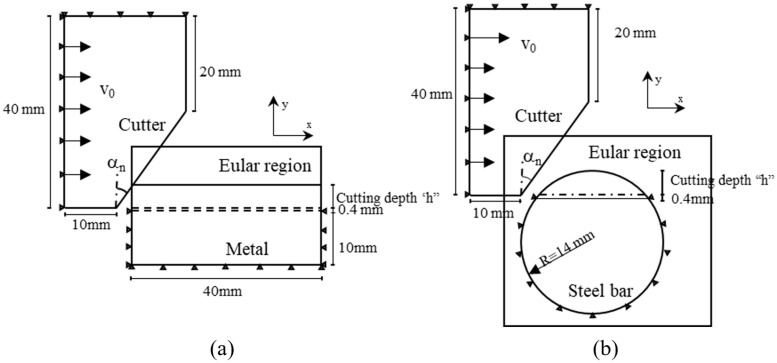
Negative rake angel cutter cutting finite element model diagram: (**a**) 2D model of orthogonal cutting with negative rake angel cutter; (**b**) 2D model of cutting circular cross-section steel bars with negative rake angle cutter.

The numerical model uses the CEL method of Abaqus to simulate the orthogonal cutting and cutting of metal with a circular cross-section with negative rake angle cutters. Since the total cutting time is very short, adiabatic stress analysis is used. A schematic diagram of the orthogonal cutting model of negative rake angle cutters is shown in Figure 1a, and a schematic diagram of the circular cross-section steel bar cutting using negative rake angle cutters is shown in Figure 1b. The CEL method can only be used in 3D models; therefore, the model width is set to 0.001 m, and the lateral displacement is constrained. The horizontal velocity is set to the cutting velocity, and the vertical velocity is set to 0. The cutting depth is set to *h*. In the pile cutting project of Shenyang Metro Line 4, the advancement speed during pile cutting was approximately 10~20 mm/min, the cutter head speed was approximately 1~2 r/min, and there were 2~3 cutters on the same cutting trajectory, so the cutting depth was approximately 1.6~10 mm each time. Therefore, different cutting depths of 2 mm, 4 mm, 6 mm, and 8 mm are considered in the model, and the cutting speed is set to 0.5 m/s. At the same time, the displacement of the boundary below the cutting depth of the workpiece or steel bar, *h* + 0.4 mm, is constrained. The radius of the steel bar is 14 mm. Considering that bentonite and foaming agent are continuously injected in front of the cutter head during shield advancement, and to simplify the analysis conditions, the friction coefficient of the contact surface between the cutter and the steel bar is considered 0. The slag temperature during pile cutting is about 50 °C, so, the initial temperature is set to 50 °C (323.15 K).

This study focuses on the cutting force of the cutter on circular cross-section steel bars under different cutting depths and different negative rake angles. The contact force between the bottom of the cutter and the steel bar is ignored, and only the horizontal and vertical forces on the negative rake angle surface are extracted as the cutting force from the numerical simulation. The main affected part of the Euler region has a mesh size of 0.5 mm, and an eight-node, reduced integration, thermal solid element is used in this region. The minimum mesh size of the cutter is 1 mm, and the eight-node, reduced integration solid element is used in this region.

Since the composition and mechanical properties of hot-rolled ribbed steel bar 335 (HRB335) and HRB400 are similar to those of No. 45 steel, which is also a carbon structural steel, the numerical simulation of cutting steel bars with a shield cutter in this study uses the Johnson–Cook model and the parameters of the material model corresponding to No. 45 steel [20]. Therefore, the Johnson–Cook plastic model and the Johnson–Cook damage model are used in this study, and the corresponding model parameters of No. 45 steel are used as parameters for the steel bar to simulate its mechanical properties. The cutter alloy is tungsten carbide steel, and the linear elastic model [20] is used. The specific material parameters are set as shown in Table 1, Table 2 and Table 3.

### 2.2. Slip Plane Analysis in the Initial Stage of Orthogonal Cutting

**Figure 2:**
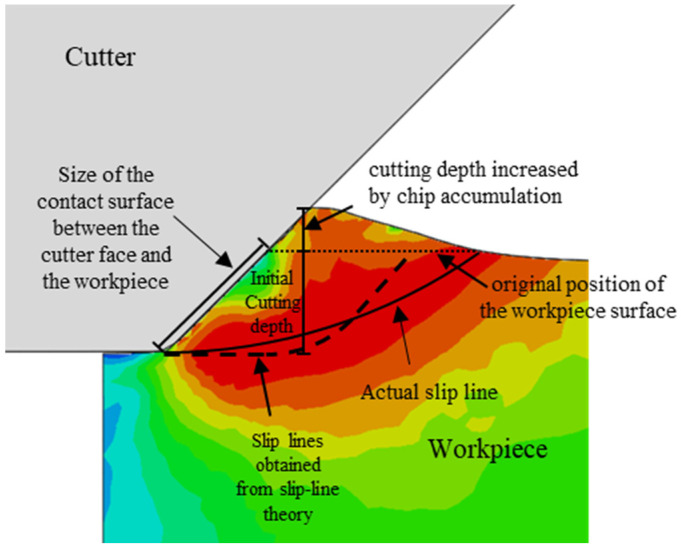
Stress cloud diagram and slip line in the initial stage of orthogonal cutting (without considering the influence of chip accumulation).

**Figure 3:**
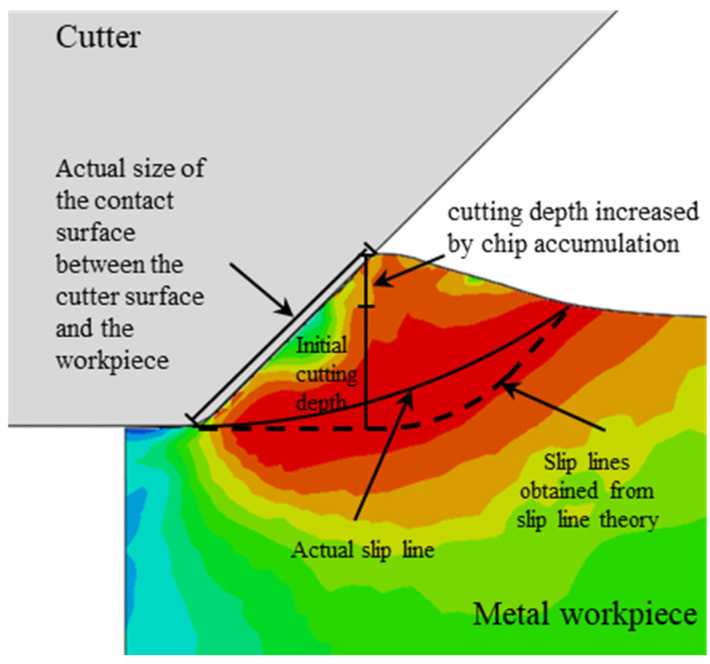
Stress cloud diagram and slip line in the initial stage of orthogonal cutting (considering the influence of chip accumulation).

The Mises stress cloud diagram obtained by numerical simulation is shown in Figure 2 and Figure 3, when the cutter has a negative rake angle of 45 degrees. As the cutting progresses, the metal chips begin to accumulate in front of the cutter. The original horizontal surface of the workpiece in front of the cutter becomes an inclined surface (see Figure 2), which continues to increase as the cut progresses. The actual contact size between the cutter surface and the workpiece increases accordingly, and the size of the *v* slip plane also gradually increases as the cutting progresses. Based on the slip line theory [42], the slip lines in Figure 2 and Figure 3 are shown as dashed lines according to the size of the contact surface between the cutter and the workpiece with and without considering the influence of chip accumulation, respectively. It can be observed that the slip plane drawn according to the slip line theory is closer to the slip plane in the numerical simulation when the influence of chip accumulation on the actual contact range between the cutter and the workpiece is taken into consideration.

### 2.3. Analysis of Cutting Force and Cutting Slip Plane

**Figure 4:**
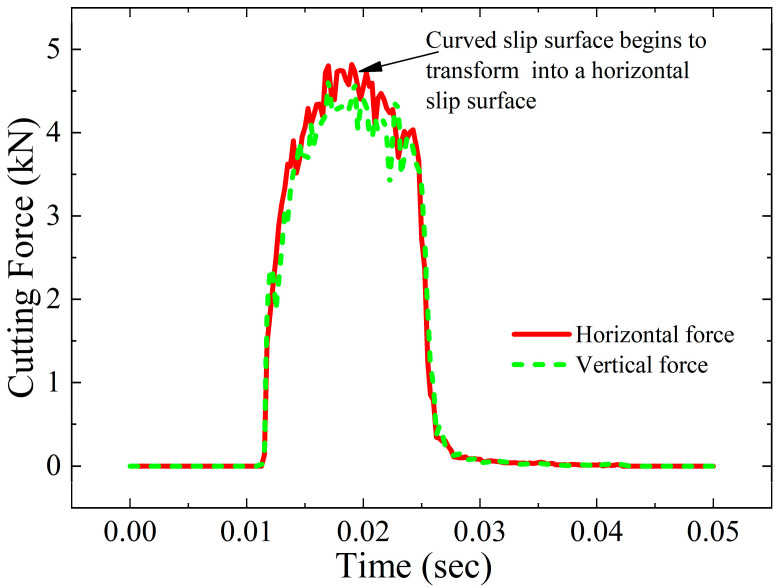
Variation in cutting force for 45° negative rake angle and cutting depth 2 mm with time.

**Figure 5:**
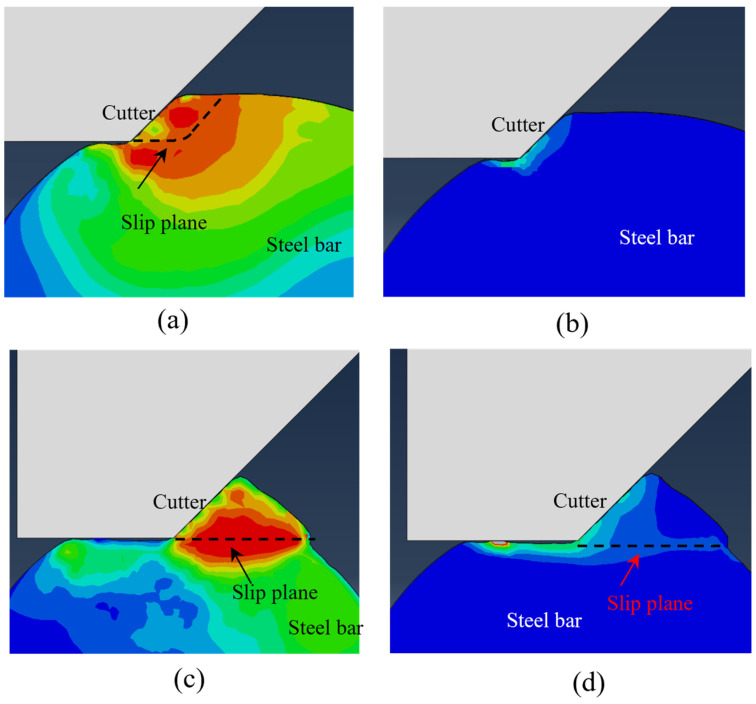
Equivalent stress cloud diagram and equivalent plastic strain cloud diagram before and after the slip surface transformation. (**a**) Mises stress cloud diagram in multi-segment slip plane. (**b**) Equivalent plastic strain cloud diagram in multi-segment slip plane. (**c**) Mises stress cloud diagram in horizontal slip plane. (**d**) Equivalent plastic strain in horizontal slip plane.

Figure 4 shows the change in cutting force of a cutter with a negative rake angle of 45 degrees and a cutting depth of 2 mm with time. As the cutter starts to cut the steel bar, the horizontal cutting force and vertical thrust increase rapidly. After the peak value is reached, the cutting force continues to decrease and returns to 0 after the cutting is completed. The Mises stress cloud diagram and equivalent plastic strain cloud diagram of the steel bar before and after the slip plane transformation obtained by numerical calculation (see Figure 5) show that at the beginning of cutting, the slip plane is close to the slip line in the initial stage of orthogonal cutting, and the generated equivalent plastic strain is also mainly concentrated in a small area in front of the negative rake angle cutter (see Figure 5a,b). As the cutting displacement increases, the slip plane is transformed into a horizontal slip plane due to the size limitation of the circular cross-section and the influence of constraint conditions (see Figure 5c,d). Under this condition, the horizontal cutting force and vertical thrust reach the maximum value. When the cutting displacement continues to increase, the circular cross-section steel bar deforms along the horizontal shear surface and finally reaches failure, and a single cutting is completed.

## 3. Cutting Model

By analyzing the numerical results of the orthogonal 2D metal cutting with negative rake angle cutters in Section 2, it can be observed that the boundary points of the slip lines obtained by considering the influence of chip accumulation according to the slip line theory are closer to the boundary points (slip planes) of the arc slip lines in the numerical simulation results. As the accumulated chips increase, the size of the slip plane changes accordingly. When the cutter cuts a circular cross-section, the slip plane changes from a curved slip plane to a horizontal slip plane under the influence of constraints, and the cutting force reaches its maximum value at this moment. Based on this, a model for calculating the cutting force when cutting a circular 2D cross-section with a negative rake angle cutter is proposed, and the cutting force is calculated based on the resultant force on the slip plane.

### 3.1. Cutting Slip Line Model

This model considers the influence of chip accumulation and determines the intersection point of the actual arc slip plane on the surface of the steel bar with a circular cross-section based on the cutter position and slip line theory. The actual arc slip plane is then determined, and the maximum yield stress on the slip plane is calculated in combination with the Johnson–Cook model to determine the magnitude of the cutter cutting force.

#### 3.1.1. Slip Lines Based on Slip Line Theory

**Figure 6:**
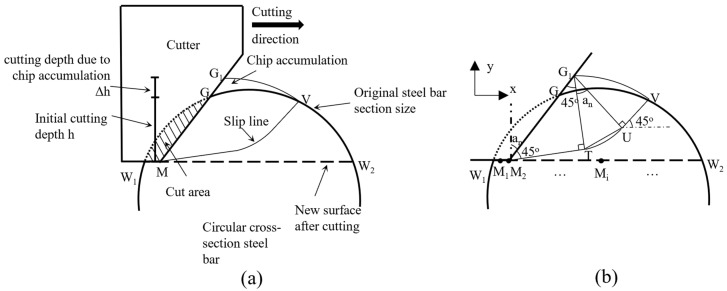
Schematic diagram of slip lines based on slip line theory. (**a**) Schematic diagram of the slip line. (**b**) Detailed diagram of slip line segment relationship.

The slip line obtained according to the slip line theory is shown in Figure 6. The size of the slip line is affected by the chip accumulation in front of the cutter, and it is assumed that the chip accumulation area (GG_1_V area) is equal to the cut area, i.e., the area represented by the shaded W_1_ MG area in Figure 6a. The chip accumulation causes the cutting depth in front of the cutter to increase by Δh, based on the initial cutting depth h. After the cut is completed, a new surface, W_1_ W_2_, is created.

According to the slip line theory [37], the slip plane of the slip line model is composed of three segments: MT, TU, and UV (see Figure 6b). When point M moves horizontally, which corresponds to different points in Figure 6b (M_1_, M_2_, etc.), Δh increases continuously and the length of MG_1_ changes accordingly, which causes the slip line to change as well. When M is at a certain point, M_i_ (point M is at position M_1_ in Figure 6b), the size of the slip line is determined by the length of cutter contact, MG_1_. The intersection angle between MT, G_1_T, and MG_1_ is 45 degrees, so the length of MT and G_1_T can be determined based on the size of MG_1_. The points T, U, and V are determined by the position of point M, the length of the line segment MG_1_, the absolute cutter negative rake angle αn and the size of the circular cross-section. The TU segment of the slip line is obtained by drawing an arc with an angle of α_n_ counterclockwise with point G_1_ as the center and G_1_T as the radius. The UV segment starts from point U and goes along the direction of 45 degrees to the horizontal positive direction (the angle increases counterclockwise along the positive direction of the *x*-axis if it is positive, and vice versa if it is negative) to obtain the intersection point V of the slip line and the circular cross-section surface. The intersection point V of the slip line and the circular cross-section surface of the steel bar are determined by the position of M, the increase in cutting depth caused by chip accumulation, and the size of the circular cross-section.

#### 3.1.2. Arc Slide Line Model Considering the Effect of Chip Accumulation

**Figure 7:**
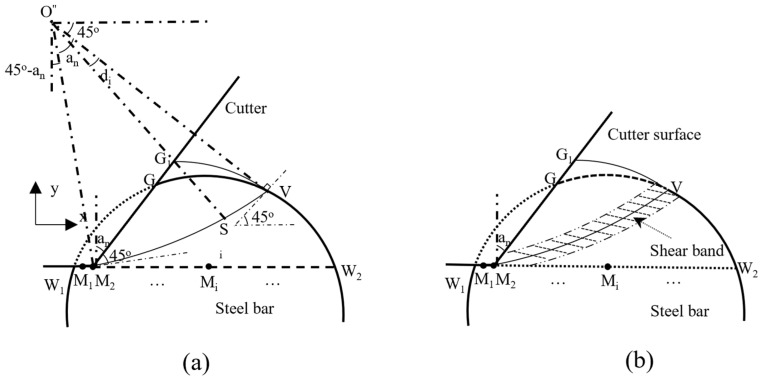
Arc slip line model. (**a**) Detailed diagram of arc slip line segment relationship. (**b**) Schematic diagram of arc slip line shear band.

From the analysis in Section 2, the position of the intersection point “V” of the actual slip line and the surface of the steel bar with a circular cross-section can be determined by using the slip line theory and considering the influence of chip accumulation. On this basis, an arc line is created as a new slip line according to the position of the cutter point M and point V (see Figure 7). Point M is located on point M_2_ of the cutting trajectory. Since point V lies on the slip line arc, and the angle between the tangent to point V and the horizontal direction is 45 degrees, M also lies on the slip line arc. The angle between the tangent at this point and the cutter surface MG_1_ is 45 degrees, so the position of the arc center point O can be determined. From the relationship shown in Figure 7a, it can be observed that the corresponding angle of the arc ∠VOM_2_ is the absolute negative rake angle of the cutter α_n_. In combination with the existing linear shear band model [39,40,41], the shear band formed in this model is assumed to be a shear band along the arc slip plane MV, as shown in the shaded region in Figure 7b.

#### 3.1.3. Calculation of Stress and Cutting Force on Arc Sliding Line

The magnitude of the flow stress on the arc slip line in Figure 7 is k. At a point S_i_ on the slip line, the average stress $\sigma_{m,si}$ is calculated as follows:

(1) $$\sigma_{m,si}=\left(1+2p_{1}\delta_{i}\right)k$$

where $\delta_{i}$ is the angle of rotation of the tangent line at point S_i_ relative to the tangent at point V. At the same time, the parameter $p_{1}$ is added to facilitate the subsequent analysis, where 0 ≤ *p*_1_ ≤ 1.

Since the tangential force is in the same direction as the slip line, the average stress on the slip line rotates 90 degrees clockwise along the direction of the tangential force. If the tangential force direction is at an angle θ to the positive direction of the *x*-axis, then the average stress (compressive stress) direction is at an angle θ-90 degrees to the positive direction of the *x*-axis. The positive direction of the *x*-axis is 0 degrees, and the angle increases counterclockwise along the *x*-axis, which is positive, and vice versa.

The angle between the flow stress, k, at the S_i_ point and the positive direction of the *x*-axis is $\left(\pi /4-\delta_{i}\right)$, and the angle between the average stress $\sigma_{m,si}$ and the positive direction of the *x*-axis is $\left(\pi /4-\delta_{i}-\pi /2\right)=-\delta_{i}-\pi /4$. If Equation (1) is combined, the horizontal stress $\sigma_{h,si}$ and vertical stress $\sigma_{v,si}$ at S_i_ point are obtained after simplification, as follows:

(2) $$\sigma_{h,si}=\left(1+2p_{1}\delta_{i}\right)k\cos\left(-\frac{\pi }{4}-\delta_{i}\right)+k\cos\left(\frac{\pi }{4}-\delta_{i}\right)$$

(3) $$\sigma_{v,si}=\left(1+2p_{1}\delta_{i}\right)k\sin\left(-\frac{\pi }{4}-\delta_{i}\right)+k\sin\left(\frac{\pi }{4}-\delta_{i}\right)$$

By dividing the horizontal stress $\sigma_{h,si}$ and the vertical stress $\sigma_{v,si}$ by k, the normalized horizontal stress and the vertical stress can be obtained as follows:

(4) $${\bar{\sigma }}_{h,si}=\left(1+2p_{1}\delta_{i}\right)k\cos\left(-\frac{\pi }{4}-\delta_{i}\right)+\cos\left(\frac{\pi }{4}-\delta_{i}\right)$$

(5) $${\bar{\sigma }}_{v,si}=\left(1+2p_{1}\delta_{i}\right)k\sin\left(-\frac{\pi }{4}-\delta_{i}\right)+\sin\left(\frac{\pi }{4}-\delta_{i}\right)$$

The normalized horizontal stress and the normalized vertical stress on the arc sliding surface are integrated to obtain the normalized horizontal cutting force and the normalized vertical cutting force, as follows:

(6) $${\bar{f}}_{h,sl,i}=\int_{0}^{\alpha n}\left[\left(1+2p_{1}\delta \right)\cos\left(-\frac{\pi }{4}-\delta \right)+\cos\left(\frac{\pi }{4}-\delta \right)\right]r^{\prime }d\delta \;\begin{array}{c} {\bar{f}}_{h,sl,i}=r^{\prime }\sqrt{2}\left[\sin\left(\alpha_{n}\right)+\left(\alpha_{n}-1\right)p_{1}\sin\left(\alpha_{n}\right)+\left(1+\alpha_{n}\right)p_{1}\cos\left(\alpha_{n}\right)-p_{1}\right] \end{array}$$

(7) $${\bar{f}}_{v,sl,i}\int_{o}^{\alpha n}\left[\left(1+2p_{1}\delta \right)k\sin\left(-\frac{\pi }{4}-\delta \right)+k\sin\left(\frac{\pi }{4}-\delta \right)\right]r^{\prime }d\delta \;\begin{array}{c} {\bar{f}}_{v,sl,i}=r^{\prime }k\sqrt{2}\left[\cos\left(\alpha_{n}\right)-\left(\alpha_{n}+1\right)p_{1}\sin\left(\alpha_{n}\right)+\left(\alpha_{n}-1\right)p_{1}\cos\left(\alpha_{n}\right)+p_{1}-1\right]\; \end{array}$$

where ${\bar{f}}_{h,sl,i}$ is the normalized horizontal cutting force obtained from the arc slip line, ${\bar{f}}_{v,sl,i}$ is the normalized vertical cutting force obtained from the arc slip line, and $\alpha_{n}$ is the absolute negative rake angle of the cutter.

It is assumed that the steel bar surface below the cutting depth is constrained, i.e., no horizontal and vertical displacement occurs. From the numerical simulation results, it is concluded that under the influence of the constraint conditions, the final slip plane changes from a curved slip plane to a horizontal slip plane, and the horizontal cutting force reaches the maximum value at the moment of the change. On this basis, the normalized horizontal cutting force ${\bar{f}}_{h,sl,i}$ obtained by the slip line model when M is located at M_i_ must be compared with the normalized cutting force ${\bar{f}}_{h,miw2}$ obtained at the corresponding horizontal shear surface M_i_W_2_ (i.e., M_i_W_2_ length $s_{miw2,i}$), and the smaller value in ${\bar{f}}_{h,sl,i}$ and ${\bar{f}}_{h,miw2}$ is taken as the horizontal cutting force at that point. Therefore, the final normalized horizontal cutting force and the normalized vertical cutting force at this point are calculated as follows:

(8) $$\left\{\begin{array}{c} {\bar{f}}_{h,mi}=min\left({\bar{f}}_{h,sl,i},{\bar{f}}_{h,miw2,i}\right)\\ {\bar{f}}_{v,mi}={\bar{f}}_{h,mi}\cdot \;{\bar{f}}_{v,sl,i}/{\bar{f}}_{h,sl,i} \end{array}\right.$$

where ${\bar{f}}_{h,mi}$ and ${\bar{f}}_{v,mi}$ are the normalized horizontal and vertical tangential force, respectively, calculated when M is located at point M_i_. ${\bar{f}}_{h,sl,i}$ and ${\bar{f}}_{v,sl,i}$ are the normalized horizontal and vertical cutting force, respectively, obtained according to the arc slip line.

### 3.2. Increased Cutting Depth

**Figure 8:**
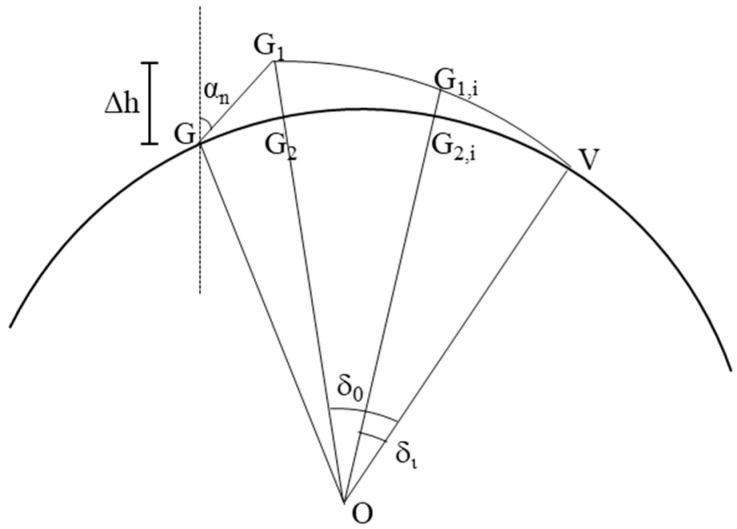
Schematic diagram of chip accumulation.

The model assumes that the area of the front accumulation of the negative rake angle (the area of the chip accumulation GG_1_V in Figure 7) is equal to the area of the cutter scanning the circular cross-section (the area of the arc W_1_MG, the shaded region in Figure 7). Since points V and G are located on the arc, assuming that the accumulation thickness ∆h is known, the position of point G and the absolute negative rake angle *α_n_* can be determined according to the model to calculate the length of GG_1_ and the position of point G_1_, as shown in Figure 8.

For the calculation, the points G, G_1_, and V are first connected to the center of the circle O. Then, G_1,i_, at any point on arc VG_1_, is connected to the center of the circle. The intersection points of line segment G_1_O, line segment G_1,i_O, and arc GV are referred to as G_2_ and G_2,I_, respectively. The angles formed by the line segment G_1_O, line segment G_1,i_O, and line segment VO are $\delta_{0}$ and $\delta_{i}$ respectively. Assuming that the length of the line segment G_1_G_2_ is $l_{G_{1}G_{2}}$ and the length of the line segment G_1,i_ G_2,i_ is $l_{G_{1,i}G_{1,i}}$, the following relationship can be obtained:

(9) $$\frac{l_{G_{1}G_{2}}}{l_{G_{1,i}G_{1,i}}}=\frac{\delta_{0}}{\delta_{i}}$$

Then, the area of the G_1_G_2_V region can be expressed as follows:

(10) $$S_{VG_{1}G_{2}}=0.5\delta_{0}\left(l_{G_{1}G_{2}}r+\frac{{l_{G_{1}G_{2}}}^{2}}{3}\right)$$

where $S_{VG_{1}G_{2}}$ is the area of the G_1_G_2_V region, and *r* is the radius of the circular cross-section. $S_{GG_{1}G_{2}}$ can be obtained by subtracting $S_{OGG_{2}}$ from the area of sector OGG_2_ and $S_{∆OGG_{1}}$ from the area of triangle OGG_1_. The area of the front accumulation of the negative rake angle can be calculated as follows:

(11) $$S_{GG_{1}V}=S_{VG_{1}G_{2}}+S_{GG_{1}G_{2}}$$

To calculate ∆h, the initial $∆h_{ini,max}$ and $∆h_{ini,min}$ must be calculated. $∆h_{ini,max}=2S_{WM_{1}G}/l_{GV_{,ini}}$, where the cutter sweeps the circular cross-section area $S_{WMG}$, $l_{GV_{,ini}}$ is the length of arc GV calculated when ∆h = 0, and $∆h_{ini,min}$ = 0. Based on the relationship that the area GG_1_V accumulates before the negative rake angle is equal to the area of the circular cross-section swept by the cutter, the different iteration is used to obtain ∆h.

### 3.3. Johnson–Cook Model

The Johnson–Cook model can take into account the influence of the plastic strain, damage, and temperature change of the material. The model can be expressed as follows:

(12) $$\bar{\sigma }=\left(1-D\right)\left[A+B{\left({\bar{\varepsilon }}^{pl}\right)}^{n}\right]\left[1+C\ln\frac{{\dot{\bar{\varepsilon }}}^{pl}}{{\dot{\varepsilon }}_{0}}\right]\left[1-{\left(\frac{T-T_{r}}{T_{m}-T_{r}}\right)}^{m}\right]$$

(13) $${\bar{\varepsilon }}_{f}^{pl}=\left[d_{1}+d_{2}exp\left(-d_{3}\eta \right)\right]\left[1+d_{4}\ln\left(\frac{{\dot{\bar{\varepsilon }}}^{pl}}{{\dot{\varepsilon }}_{0}}\right)\right]\left[1+d_{5}\frac{T-T_{r}}{T_{m}-T_{r}}\right]$$

(14) $$D=\sum \frac{d{\bar{\varepsilon }}^{pl}}{{\bar{\varepsilon }}_{f}^{pl}}$$

where *A*, *B*, *C*, *m*, and *n* are the material parameters of the Johnson–Cook plasticity model; *d*_1_~*d*_5_ are the material parameters for the Johnson–Cook damage model; $\bar{\sigma }$ is the equivalent stress; ${\bar{\varepsilon }}^{pl}$ is the cumulative equivalent plastic strain; ${\dot{\bar{\varepsilon }}}^{pl}$ is the equivalent plastic strain rate; ${\dot{\varepsilon }}_{0}$ is the reference strain rate; $d{\bar{\varepsilon }}^{pl}$ is the equivalent plastic strain increment; ${\bar{\varepsilon }}_{f}^{pl}$ is the equivalent plastic strain when the material fails; *D* is the damage value 0≤D≤1; T is the material temperature; $T_{r}$ is the critical temperature, i.e., when $T<T_{r}$, the strength of the material is not affected by temperature, and $T_{m}$ is the melting temperature of the material; and η = −p/q, where *p* is the average stress, being the same as $\sigma_{m,si}$ in Equation (1), and *q* is the *Mises* equivalent stress, which is assumed to be equal to $\bar{\sigma }$ in the calculation.

The increase in the plastic strain of the material leads to an increase in the temperature of the material, as follows [37]:

(15) $$\beta_{T}\bar{\;\sigma }d{\bar{\varepsilon }}^{pl}=\rho cdT$$

where $\beta_{T}$ is the ratio of the plastic work causing the temperature increase for the total plastic work, which is taken as 0.9; $\bar{\sigma }$ is the equivalent stress, calculated according to Equation (12); $d{\bar{\varepsilon }}^{pl}$ is the equivalent plastic strain increment; *c* is the material’s specific heat capacity; ρ is the material density; and dT  is the temperature increment.

Under plane strain conditions,

(16) $$k=\bar{\;\sigma }/\sqrt{3}$$

where k is the flow stress in Equation (1), i.e., the shear stress on the slip line, and $\bar{\;\sigma }\;$ is the equivalent stress of the material obtained from Equation (12) according to the Johnson–Cook model. Subsequently, p/q can be taken as the average value of the p/q value on the slip line segment. Then, based on Equation (1), the average value can be obtained as:

(17) $$p/q=\left(1+p_{1}\alpha_{n}\right)/\sqrt{3}$$

The Johnson–Cook model takes into account both material properties (such as hardness and strain rate sensitivity) and temperature effects, which are influenced by the microstructure and hardness of the material. These factors are integrated into the cutting force model through the Johnson–Cook model as described below:

- The Johnson–Cook model incorporates strain hardening (related to material toughness and hardness) and strain rate sensitivity, which reflects the material’s response to high-speed deformation. In materials with higher hardness, the strain hardening coefficient will be higher, indicating that the material strengthens more under deformation and requires greater cutting forces. These are reflected in the Johnson–Cook model’s parameters, such as the yield strength coefficient A, the strain hardening coefficient B, and the strain rate sensitivity coefficient C.
- The temperature effects are also modeled in the Johnson–Cook model, which accounts for how the material’s strength decreases at higher temperatures due to the heat generated during the cutting process. For materials with higher hardness, temperature increases may reduce the material’s strength, making it easier to cut at elevated temperatures, but also leading to more heat buildup and potential tool wear. The temperature-dependent term $\left[1-{\left(\frac{T-T_{r}}{T_{m}-T_{r}}\right)}^{m}\right]$ in the Johnson–Cook model accounts for the reduction in the strength of the material as temperature increases.
  The damage criterion in the Johnson–Cook model can predict the onset of damage due to the nucleation, growth, and coalescence of voids, and the yield stress decreases with damage accumulation in the Johnson–Cook model.
- The damage criterion in the Johnson–Cook model can predict the onset of damage due to the nucleation, growth, and coalescence of voids, and the yield stress decreases with damage accumulation in the Johnson–Cook model.

### 3.4. Material Strain Rate

#### 3.4.1. Relationship Between Cutting Speed and Strain Rate

The shear band model assumes that plastic deformation takes place in a narrow shear band divided by a shear plane [39], as shown in Figure 7b. The shear strain rate distribution in the shear band can be expressed by a high-order power function, as follows [40]:

(18) $$\dot{\gamma }=\left\{\begin{array}{c} {\dot{\gamma }}_{s}{\left(1-\frac{\bar{x}}{\lambda }\right)}^{\omega }if\;\left(0\leq \bar{x}\leq 0.5\right)\\ {\dot{\gamma }}_{s}{\left(1+\frac{\bar{x}}{1-\lambda }\right)}^{\omega }if\;\left(-0.5\leq \bar{x}\leq 0\right) \end{array}\right.$$

where ${\dot{\gamma }}_{s}$ is the shear strain rate on the slip plane, $\dot{\gamma }$ is the shear strain rate at distance *x* from the shear plane in the shear band, $\bar{x}=x/h_{0}$, *x* is the distance from the shear plane at a point in the shear band, and $h_{0}$ is the thickness of the shear band. To simplify the calculation, λ is assumed to be equal to 0.5. ω is a material-related parameter. According to the study of Shi et al. [43], ω = 4.

Taking the lower half of the shear band and integrating it, the following can be obtained:

(19) $$V_{s}=\int_{-0.5h}^{0}{\dot{\gamma }}_{s}{\left(1+\frac{x}{0.5h}\right)}^{\omega }dx=\frac{0.5{\dot{\gamma }}_{s}}{\omega +1}h_{0}$$

$h_{0}$ can be calculated as follows [40]:

(20) $$h_{0}=l_{s}/C_{0}$$

where, $V_{s}$ is the shear velocity on the slip plane; and $C_{0}$ is the ratio of the length of the slip plane $l_{s}$ to the thickness of the shear band $h_{0}$.The range of $C_{0}$ in the existing shear band model is 2 to 10 [39]. $l_{s}$ is the length of the shear plane in the existing shear band model. In this model, $l_{s}$ is the arc length of slip line in Figure 7. Combining Equations (19) and (20), the following relationship can be obtained:


(21) $${\dot{\gamma }}_{s}=\frac{2\left(\omega +1\right)V_{s}}{l_{s}}C_{0}$$

Assuming that the shear velocity, $V_{s}$, and the horizontal cutting speed, $V_{h}$, satisfy the relationship, the following relationship can be written as:

(22) $$V_{s}/V_{h}=l_{s}/l_{s,x}$$

where $l_{s,x}$ is the projection length of the circular slip line along the horizontal direction in Figure 7, which can be obtained based on the coordinates of points M and V. Then, according to the plane strain condition:

(23) $$\dot{\bar{\varepsilon }}=\frac{{\dot{\gamma }}_{s}}{\sqrt{3}}$$

Assuming that,

(24) $${\dot{\bar{\varepsilon }}}^{pl}=\dot{\bar{\varepsilon }}$$

where ${\dot{\bar{\varepsilon }}}^{pl}$ is the equivalent plastic strain rate and $\dot{\bar{\varepsilon }}\;$ is the equivalent strain rate. By substituting Equation (21) with Equation (24), the latter can be rewritten as follows:

(25) $${\dot{\bar{\varepsilon }}}^{pl}=\frac{1}{\sqrt{3}}\frac{2\left(\omega +1\right)V_{h}}{l_{s,x}}C_{0}$$

Based on Equation (25), the horizontal projection length $l_{s,x}$ and the horizontal cutting speed $V_{h}$ of the slip line at different M points (M_1_, M_2_, … M_i_ in Figure 7) in the circular slip line model, the equivalent plastic strain rate ${\dot{\bar{\varepsilon }}}^{pl}$ at the corresponding point can be obtained.

#### 3.4.2. Determination of the Average Equivalent Plastic Strain Rate

Since the corresponding shear plane near the end of the cutting side (W_2_ point side on Figure 7) is small, the corresponding shear band thickness is small, and the obtained equivalent plastic strain rate ${\dot{\bar{\varepsilon }}}^{pl}$ is abnormally large compared to the equivalent plastic strain rate at the middle position. Therefore, the average value of the equivalent plastic strain rate obtained when the M point is located at the center line of the steel bar section at the beginning of cutting (the left half of Figure 7) is taken as the equivalent plastic strain rate ${\dot{\bar{\varepsilon }}}^{pl}$ required for the calculation of the maximum flow stress ${\;k}_{max}$ for Equation (16).

### 3.5. The Influence of $C_{0}$ Value on the Maximum Mises Stress

From Equation (25), it can be observed that the equivalent plastic strain rate ${\dot{\bar{\varepsilon }}}^{pl}\;$ at the slip plane is linearly related to the value of $C_{0}$ when cutting speed and slip line are determined. Since the value range of $C_{0}$ is 2~10 [39], the maximum and minimum equivalent strain rates ${\dot{\bar{\varepsilon }}}_{s,max}$ and ${\dot{\bar{\varepsilon }}}_{s,min}$ corresponding to a $C_{0}$ of 2 or 10 are taken to satisfy ${\dot{\bar{\varepsilon }}}_{s,max}/{\dot{\bar{\varepsilon }}}_{s,min}$ = 5, and a different ${\dot{\bar{\varepsilon }}}_{s,min}\;$ is taken for analysis. When ${\dot{\bar{\varepsilon }}}_{s,min}$ is 10, 100, 500, 1000, 5000, and 10,000, the corresponding maximum equivalent strain rate ${\dot{\bar{\varepsilon }}}_{s,max}$ is 50, 500, 2500, 5000, 25,000, and 50,000, and the maximum Mises relationship is calculated. In order to facilitate the calculation, p/q at point M is taken as the calculation value, where p is the pressure value and q is the equivalent stress. According to Equation (1), when p_1_ = 1, $p=\sigma_{m}=\left(1+2\alpha_{n}\right)k$, and $q=\sqrt{3}k$, where $\alpha_{n}$ takes the corresponding radian values of the absolute negative rake angles of 10, 20, 30, 40, and 50, the following relationship can be obtained:

(26) $$p/q=\left(1+2p_{1}\alpha_{n}\right)k/\sqrt{3}k=\left(1+2p_{1}\alpha_{n}\right)/\sqrt{3}$$

**Figure 9:**
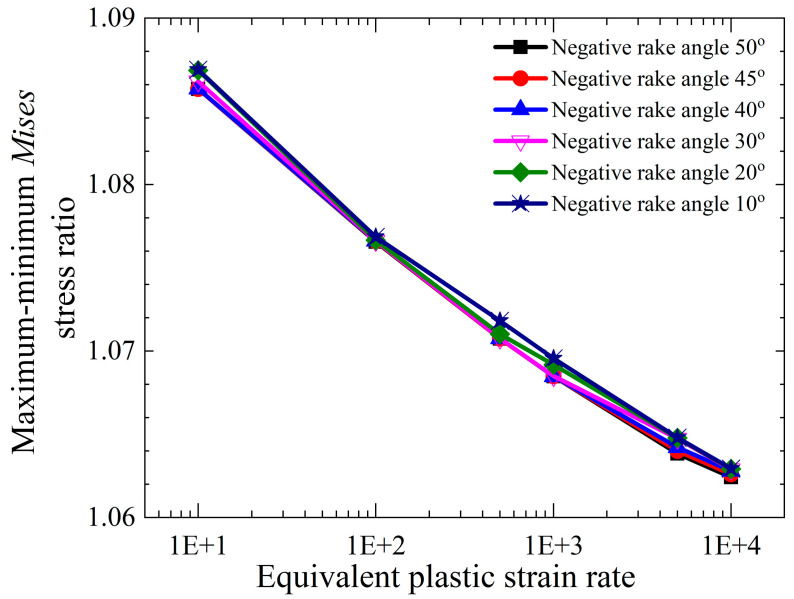
Change in maximum–minimum *Mises* stress ratio with plastic strain rate.

The maximum Mises stress ratio estimated by calculating different ${\dot{\bar{\varepsilon }}}_{s,min}$ values and the corresponding ${\dot{\bar{\varepsilon }}}_{s,max}$ values under different negative rake angle conditions is shown in Figure 9. The material parameters are shown in Table 2 and Table 3. The figure shows that $C_{0}$ has only a small influence on the maximum Mises stress, which is no more than 1.09 times the minimum Mises stress in the range of 2 to 10. Therefore, the middle value of 5.0 is taken to simplify the calculation.

### 3.6. Maximum Cutting Force Calculation

Based on the proposed model, the steps for calculating the maximum cutting force can be obtained. Firstly, the normalized horizontal and vertical cutting force ${\bar{\;f}}_{h,mi}$ and ${\bar{\;f}}_{v,mi}$, respectively, at the points M_1_, M_2_, …, and M_i_ are calculated in sequence using the slip line model. Based on the calculated normalized horizontal cutting forces, the maximum value of ${\bar{\;f}}_{h,m1},{\bar{f}}_{h,m2},{\bar{f}}_{h,m3},\ldots$ is taken as ${\bar{\;f}}_{h,max}$, and the corresponding ${\bar{f}}_{v,max}$ is determined as follows:

(27) $$\left\{\begin{array}{c} {\bar{\;f}}_{h,max}=max\left({\bar{f}}_{h,m1},{\bar{f}}_{h,m2},{\bar{f}}_{h,m3},\ldots \right)\\ {\bar{f}}_{v,max}={\bar{f}}_{v,mi} \end{array}\right.$$

where ${\bar{\;f}}_{h,max\;}$ is the normalized maximum horizontal cutting force, and ${\bar{f}}_{v,max}$ is the normalized maximum vertical cutting force. ${\bar{f}}_{v,mi}$ is the corresponding vertical force value when ${\bar{f}}_{h,mi}$ = ${\bar{\;f}}_{h,max}$

According to the horizontal projection length $l_{s,x}$ and the horizontal cutting speed $V_{h}$ of the slip line at different M points (M_1_, M_2_, … M_i_ … in Figure 7), the equivalent plastic strain rate ${\dot{\bar{\varepsilon }}}^{pl}$ at the corresponding point is obtained based on Equation (25).The average value ${\dot{\bar{\varepsilon }}}^{pl}$ of the equivalent plastic strain rate is calculated based on the plastic strain rate calculation when the M point is located at the center line of the steel bar section at the beginning of cutting (the left half of Figure 7).

Based on the Johnson–Cook elastic–plastic damage model, in Equations (12)–(15), the maximum equivalent stress $\bar{\sigma }$ is calculated, and the maximum flow stress $k_{max}$ is determined based on Equation (15). Finally, ${\bar{f}}_{h,max}$, ${\bar{f}}_{v,max}$, and k are multiplied to obtain the maximum horizontal cutting force $f_{h,max}$ and the vertical thrust $f_{v,max}$.

The specific calculation steps are as follows:

**(a)**  **Parameters for model initialization**
  - The parameters for the size of the steel bar (diameter), the cutting depth, the cutter negative rake angle, the cutting speed, the model width, and material parameters of the Johnson–Cook model are determined.
  - The average value of p/q on the slip line is determined according to Equation (17).
**(b)**  **Calculation of normalized cutting forces based on slip lines**
  i. Taking the center of the circular cross-section as the coordinate origin O, the range of the horizontal coordinate *x* of point M is determined at cutting depth z. Multiple (such as 1000) uniformly distributed sample points (M_1_, M_2_, …,M_i_) are arranged on the M cutting trajectory.
  ii. Based on point M_1_ (M is at point M_1_) and the negative rake angle, the initial length and direction of the slip line are determined based on the length of M_1_G, the negative rake angle, and the relationship between M_1_G_1_ and segments of the slip line M_1_T, TU, and UV described in Section 3.1.1. The coordinates of the points on the slip line, T, U, and V, are determined based on the size of the circular cross-section without considering the influence of chip accumulation in front of the cutter.
  iii. Based on the coordinates of point M_1_ and the corresponding coordinates of point G, the area of the circular cross-section scanned by the cutter is determined. According to the method described in Section 3.2, the increase in the cutting depth, ∆h before the cutter at this point, is solved iteratively, and point G_1_’s coordinates are determined. Then, the coordinates of the points T, U, V on the slip line are determined considering the influence of chip accumulation in front of the cutter, as shown in Figure 6.
  iv. Based on the coordinates of point V on the slip line, considering the influence of chip accumulation in front of the cutter, the coordinates of point M_1_ and the absolute negative rake angle of the cutter *α_n_*, the final normalized horizontal cutting force, and the normalized vertical cutting force at this point are determined according to the methods described in Section 3.1.2 and Section 3.1.3 (Equations (6)–(8)).
  v. The equivalent plastic strain rate ${\dot{\bar{\varepsilon }}}^{pl}$ under the slip line condition at this point is determined according to Equation (25).
  vi. For all other remaining points (M_2_, M_3_,…,M_i_), the calculation of the normalized cutting force ${\bar{f}}_{h,mi}$, ${\bar{f}}_{v,mi}$, and equivalent plastic strain rate ${\dot{\;\bar{\varepsilon }}}^{pl}$ at the corresponding positions are repeated.
  vii. The normalized horizontal cutting force ${\bar{f}}_{h,mi}$, which was determined in step B (iv) at each M_i_ point, is compared along the cutting trajectory. The maximum normalized horizontal cutting force ${\bar{f}}_{h,max}$ is obtained by taking the maximum value, and the vertical cutting force at the corresponding point of the maximum horizontal cutting force is the maximum normalized vertical cutting force ${\bar{f}}_{v,max}$, as shown in Equation (26).
**(c)**  **Calculation of maximum flow stress**
  i. Based on the equivalent plastic strain rate ${\dot{\;\bar{\varepsilon }}}^{pl}$ at each M_i_ point determined in step B, the average value of the equivalent plastic strain rate ${\dot{\;\bar{\varepsilon }}}^{pl}$ determined from the M point from the beginning of cutting to the center line of the steel bar section (the left half of Figure 7) is taken as the equivalent plastic strain rate for subsequent calculation.
  ii. Based on the average equivalent plastic strain rate ${\dot{\;\bar{\varepsilon }}}^{pl}$ determined in step C (i), the average p/q determined in step A (ii), and the material parameters determined in step A, the maximum flow stress $k_{max}$ is determined by combining the Johnson–Cook plastic damage model in Section 2.3 (Equations (12)–(16)).
**(d)**  **Calculation of maximum cutting forces**
  i. The maximum horizontal cutting force $f_{h,max}$ and vertical thrust $f_{v,max}$ can be determined by multiplying the ${\bar{f}}_{h,max}$ and ${\bar{f}}_{v,max\;}$ obtained in step B (vii) with the maximum flow stress $k_{max}$ obtained in step C (ii) and the set model width (1 mm in this paper).

## 4. Comparative Analysis of Theoretical Calculation and Finite Element Numerical Simulation Results

### 4.1. Comparison Between the Proposed Model and Numerical Simulation Results

**Figure 10:**
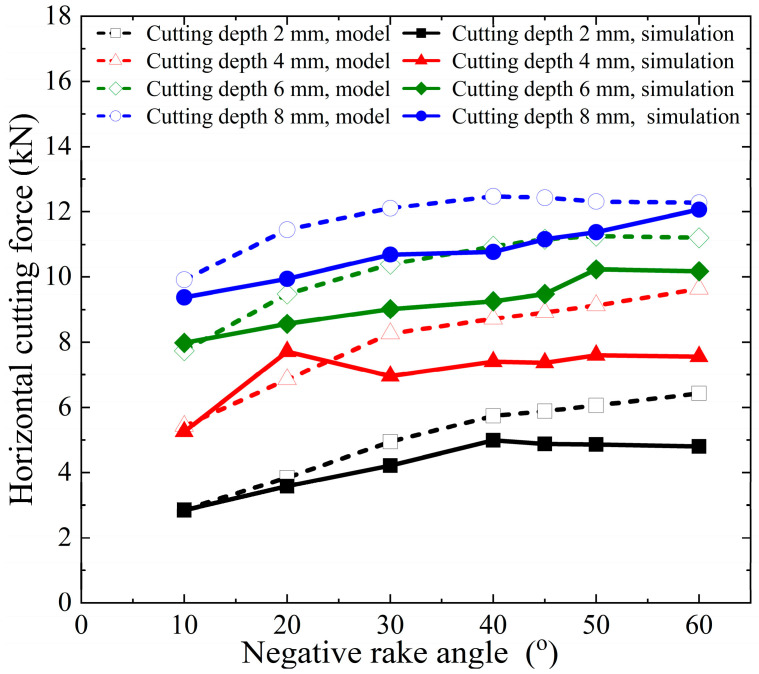
Horizontal cutting force calculation results of proposed model and numerical simulation.

**Figure 11:**
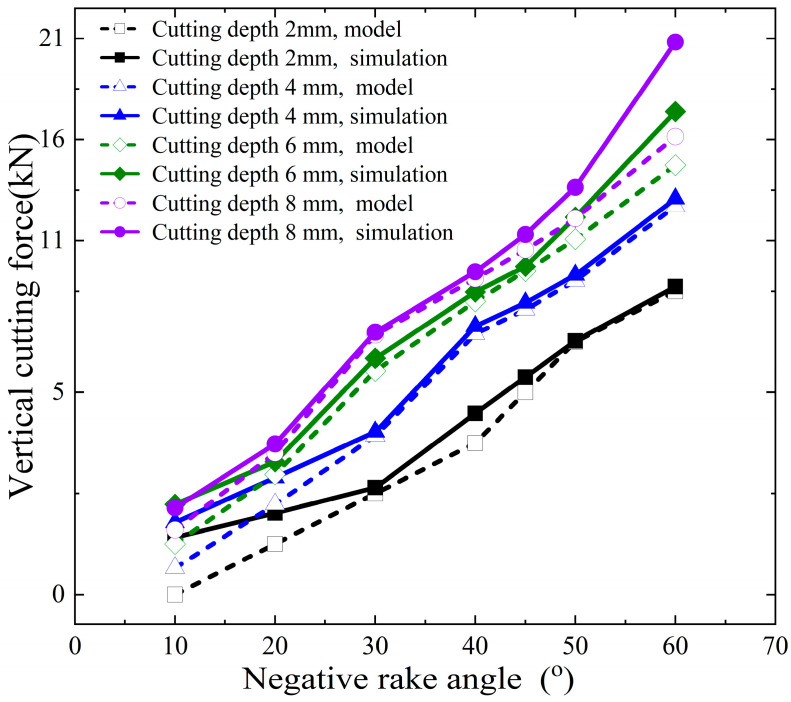
Vertical cutting force calculation results of proposed model and numerical simulation.

**Figure 12:**
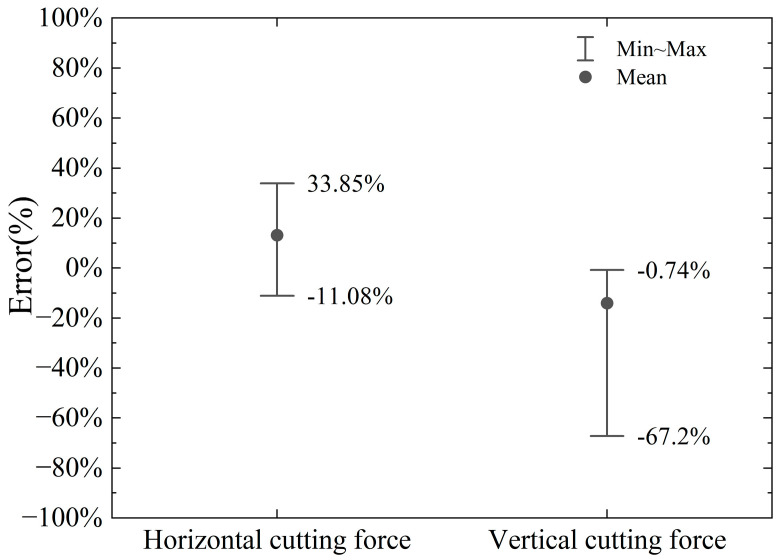
Error range in calculation of vertical and horizontal cutting force by proposed model compared to numerical simulation.

The horizontal cutting force and vertical cutting force calculated by the model and numerical simulation in this paper are shown in Figure 10 and Figure 11. The errors of the horizontal cutting force and vertical cutting force calculated by the proposed model compared to the numerical simulation results are shown in Figure 12.

The proposed model’s calculated horizontal cutting force shows an error range of −11.08% to +33.85% when compared to the numerical simulation results. In contrast, the vertical cutting force exhibits a larger error range, spanning from −67.20% to −0.74%. These results indicate that the vertical force calculations are less accurate compared to the horizontal force calculations.

The primary source of these errors lies in the fact that finite element numerical analysis can dynamically calculate the interactions between various parts (divided units) of the entire simulation object, considering given parameters and boundary conditions. It also accounts for the stress and strain states on the slip plane within the shear band and even at different points across the entire model, as illustrated in Figure 5. In contrast, the model proposed in this paper simplifies the analysis object. On one hand, the force on the cutter is determined by the stress and strain state of the local slip plane and the assumed shear band. On the other hand, the theoretical model assumes a uniform stress state at every point on the slip plane. As the cutting depth increases, the stress localization becomes more pronounced, and the material experiences varying degrees of plastic deformation at different points. This can lead to increased cutting forces, which are not fully predicted by the simplified model. In addition, the boundary conditions used in the model may not fully capture the effects of increased material interaction and cutter engagement at larger depths, leading to discrepancies in the predicted forces. Numerical simulations, by contrast, can better account for these dynamic changes in engagement and the resulting force distributions. The proposed model considers chip accumulation in front of the cutter, which plays a significant role in determining the cutting force, especially at larger depths. However, the model assumes a simple relationship between the chip formation and the cutting force, which may not fully account for the complex dynamics of chip flow and heat generation during deeper cuts.

The discrepancies observed in Figure 10 and Figure 11 are primarily due to the simplified nature of the proposed cutting force model, which does not fully capture the complexities of material behavior, thermal effects, strain distribution, and chip dynamics at larger cutting depths. While the model provides a reasonable estimate of the cutting force in the initial cutting stages, it is less accurate as the cutting depth increases, when more intricate interactions and non-linear effects come into play.

### 4.2. The Effect of Increased Cutting Depth on the Calculation of Cutting Force

**Figure 13:**
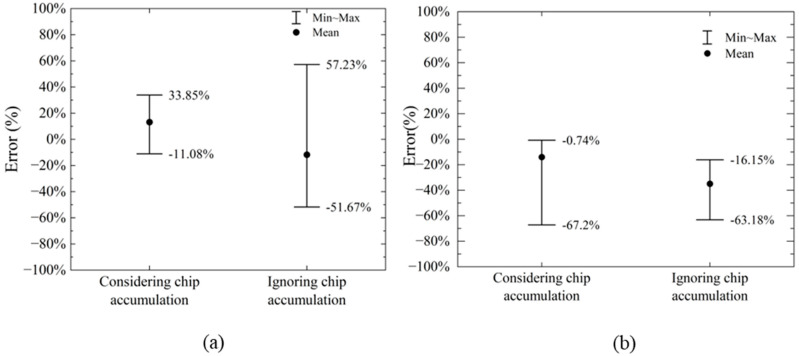
Error range considering chip accumulation: (**a**) calculation of horizontal cutting force; (**b**) calculation of vertical cutting force.

The error between the calculated horizontal cutting force and the numerical simulation results without considering the influence of chip accumulation in front of the cutter is shown in Figure 13.

From Figure 13a, it can be observed that the horizontal cutting force calculated using the proposed model, which accounts for chip accumulation in front of the cutter, closely matches the results from numerical simulations. However, when the chip accumulation effect is ignored, the error in the horizontal cutting force calculated by the model ranges from −51.67% to 57.23%. Therefore, factoring in chip accumulation results in horizontal cutting force calculations that are more accurate compared to when chip accumulation is not considered. Regarding the vertical cutting force (see Figure 13b), the error range for the model’s results, without considering chip accumulation, is between −63.18% and −16.15%. This is not significantly different from the error range of −67.20% to −0.74% observed when chip accumulation is taken into account.

### 4.3. Effect of Parameter p_1_

**Figure 14:**
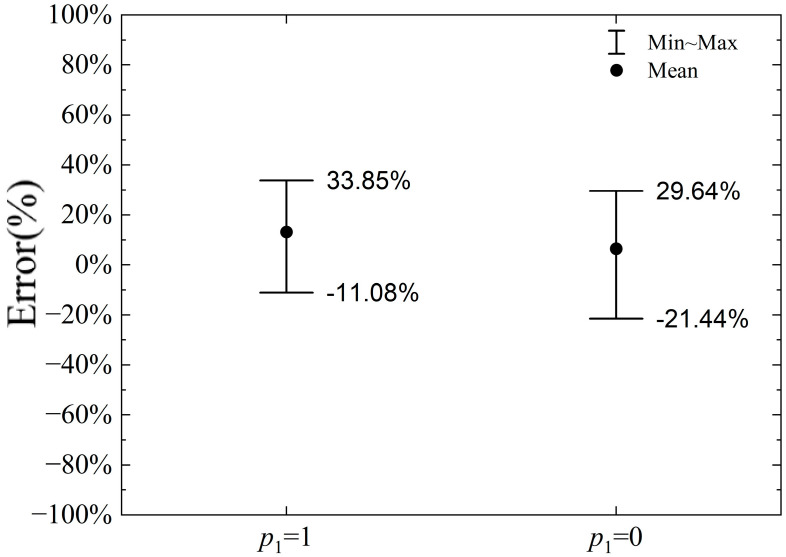
Error range of horizontal cutting force when *p*_1_ = 1 or 0.

**Figure 15:**
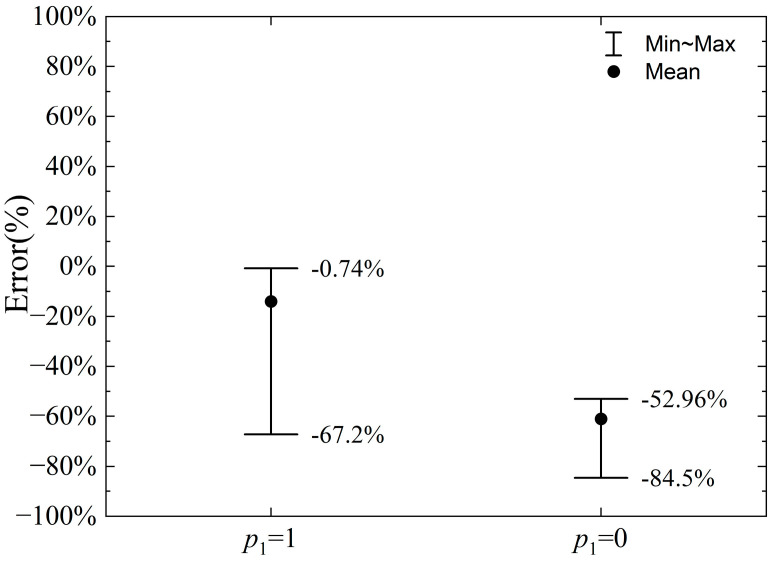
Error range of vertical cutting force when *p*_1_ = 1 or 0.

To analyze the parameter p_1_, two cases are considered: (i) the pressure on the slip line changes according to the slip line theory, i.e., parameter p_1_ = 1 in Equation (1); and (ii) the pressure on the slip line does not change with the rotation of the slip line tangent, and the average stress $\sigma_{m}=k$ is maintained, i.e., parameter p_1_ = 0 in Equation (1). The error in the calculation of the horizontal and vertical cutting forces by the proposed model compared to the numerical calculation result is shown in Figure 14 and Figure 15, respectively, when parameter p_1_ = 1 and p_1_ = 0.

As shown in Figure 14, the error range for the horizontal cutting force calculation is −11.08% to +27.42% when p_1_ = 1 and −21.44% to +29.64% when p_1_ = 0. Therefore, the error range for p_1_ = 0 is slightly larger than that for p_1_ = 1. However, regardless of whether p_1_ is set to 1 or 0, the horizontal cutting force calculated by the proposed model closely aligns with the results from the numerical simulation. Since the horizontal cutting force is determined by the horizontal stress component at each point on the slip line, and the average stress on the slip line decreases from point V to point M_1_ along the slip line in Figure 7, the influence on the horizontal force diminishes. As a result, the change in p_1_ has a relatively small impact on the calculated horizontal cutting force.

As shown in Figure 15, the error range for the vertical cutting force calculation is -67.20% to −0.74% when *p*_1_ = 1, while the error range when *p*_1_ = 0 is −84.50% to −52.96%, indicating a significant increase in the error range. This increase is primarily due to the fact that, when *p*_1_ = 1, the vertical cutting force calculated by the proposed model tends to be smaller than the numerical simulation results across different cutting depths and negative rake angle values. Since the average stress calculated for *p*_1_ = 0 is lower, the corresponding vertical force is smaller, leading to a further increase in the maximum absolute error of the vertical cutting force calculation.

### 4.4. Comparison Between the Proposed Model and Cutting Simulation Results Under Other Steel Parameter Conditions

In order to evaluate the applicability of the proposed model to other steel material parameters, the material parameters of American Iron and Steel Institute 1045 (AISI1045) [44], AISI4340 [45], and AISI304 [46] were selected to compare the cutting force results calculated by this model with the numerical simulation results. AISI 1040 is a medium-carbon steel that contains approximately 0.40% carbon. The microstructure of AISI 1040 consists mainly of ferrite and pearlite, giving it moderate hardness and strength. The strain hardening coefficient (B) and the strain rate sensitivity (C) in the Johnson–Cook model are moderate due to the relatively lower carbon content and the ferrite–pearlite structure. AISI 4340 is a high-strength, low-alloy steel that contains higher amounts of nickel, chromium, and molybdenum, which provide excellent hardenability and toughness. AISI 4340’s microstructure can consist of bainite, martensite, or a mixture of both, especially after heat treatment, giving it much higher strength and hardness than AISI 1040. The strain hardening (B) and strain rate sensitivity (C) coefficients in the Johnson–Cook model for AISI 4340 are higher due to its high-strength microstructure (such as martensite and bainite). AISI 304 is an austenitic stainless steel known for its excellent corrosion resistance, high ductility, and good weldability. It contains approximately 18% chromium and 8% nickel, which gives it an austenitic structure. The material is relatively softer than AISI 4340 but harder than AISI 1040, with a typical Brinell hardness in the range of 150–190. The strain hardening coefficient (B) is moderate for AISI 304, but it is more ductile than AISI 4340, meaning it will deform more easily under applied cutting forces. The strain rate sensitivity (C) is also moderate, as AISI 304 shows higher resistance to deformation at high strain rates (cutting speeds) compared to AISI 1040 but not as much as AISI 4340. The temperature dependence plays an important role in the model, as AISI 304’s strength is significantly affected by elevated temperatures, especially in high-speed cutting operations. The material’s ductility decreases with temperature rise, affecting cutting force predictions.

**Figure 16:**
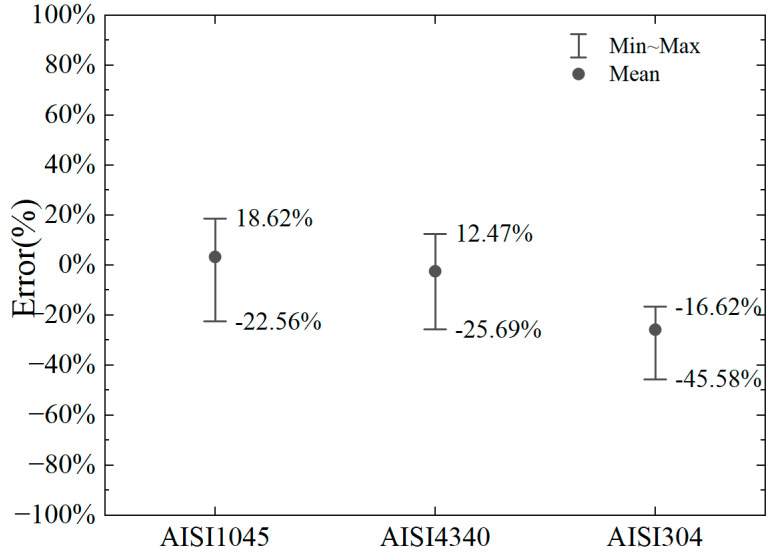
Error range in horizontal cutting force for different material parameters.

**Figure 17:**
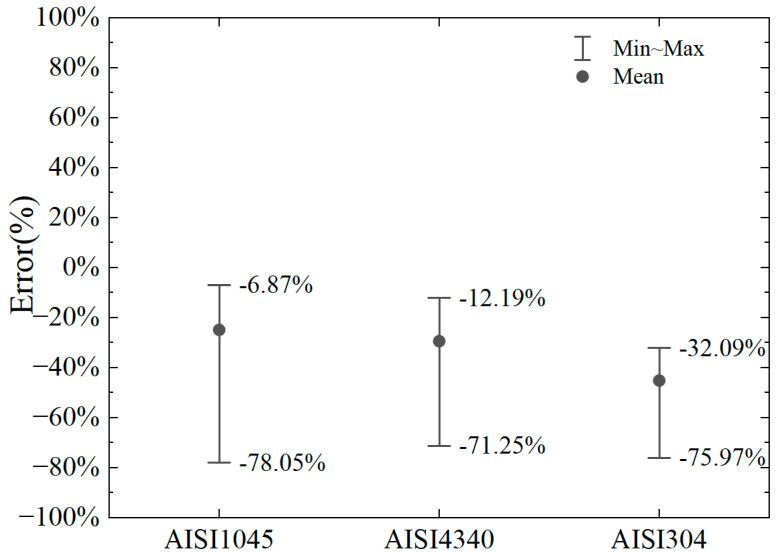
Error range in vertical cutting force for different material parameters.

The numerical model is the same as the previous two-dimensional cutting model, taking into account the conditions of cutting depths of 2 mm, 4 mm, 6 mm, and 8 mm and considering the absolute negative rake angles of 10°, 30°, and 50° at the same cutting depth. The calculation errors are shown in Figure 16 and Figure 17.

It can be observed that the error in the horizontal cutting force calculated by the proposed model, compared to the numerical results, is small for AISI1045 and AISI4340 steels. For AISI1045, the error range of the horizontal cutting force calculation is −22.56% to +18.62%. For AISI4340, the error range is −25.69% to +12.47%. For AISI304, the error range is −45.58% to −16.62%. Based on Equations (12)–(14) and comparing the material parameters of AISI304 [46] with those of No. 45 steel [20], AISI1045 [44], and AISI4340 [45], it can be observed that the temperature softening parameter (C) of yield stress in the Johnson–Cook model for AISI304 and parameter (d_5_) for temperature-affected equivalent plastic strain at material failure are significantly lower than those of the other materials.

Additionally, the equivalent plastic strain at initial material failure (d_1_) is considerably larger for AISI304 compared to the others, which affects damage development under the same plastic strain. These differences in parameters highlight the high-temperature resistance, toughness, and good processing performance of AISI304 relative to other common steels. Due to these variations, the error in the proposed model’s calculation of the cutting force for AISI304 is larger than for other steels, with the calculated results being smaller than the numerical results. However, for common steels like No. 45 steel, AISI1045, and AISI4340, the proposed model can more accurately calculate the magnitude of the horizontal cutting force when the negative rake angle is between 10° and 50°.

### 4.5. Effect of Friction Coefficient on Numerical Results

**Figure 18:**
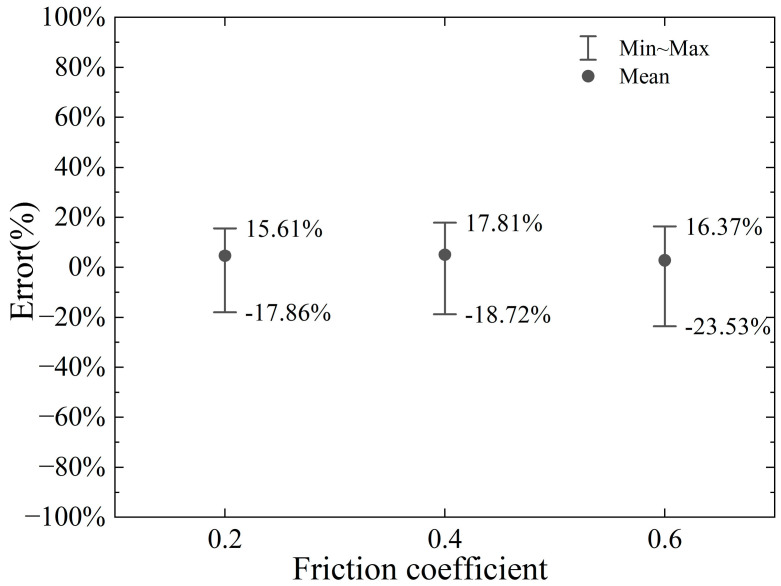
Error range in horizontal cutting force for different friction coefficients.

**Figure 19:**
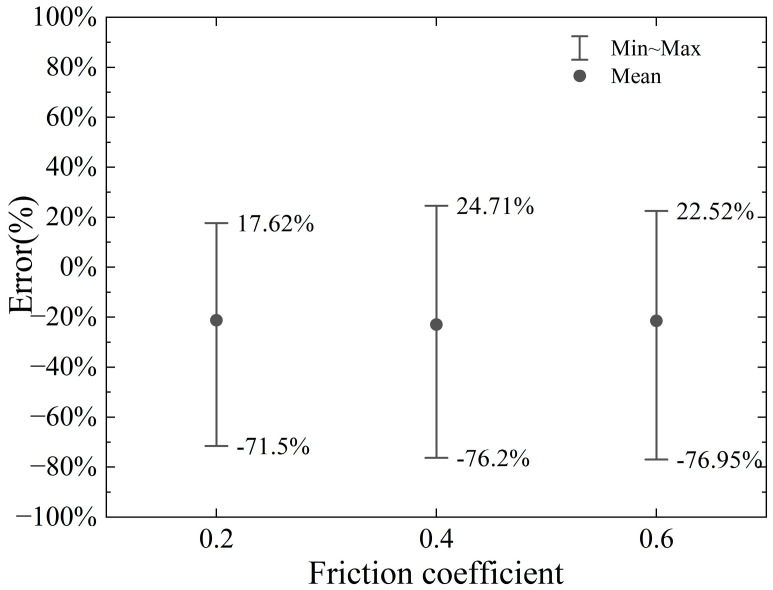
Error range in vertical cutting force for different friction coefficients.

Wang Fei [20] used a friction coefficient of 0.2 (dynamic friction coefficient 0.15) when analyzing the cutting force of a cutter cutting steel bars. Given that bentonite and foam agents are injected ahead of the cutter head during the propulsion process to minimize friction between the cutter and the soil, as well as the reinforced concrete in front, a low friction coefficient range of 0.2 to 0.6 is assumed. The numerical analysis results for friction coefficients of 0.2, 0.4, and 0.6 are compared with the proposed model’s calculations, which do not account for the influence of the friction coefficient. The resulting errors are illustrated in Figure 18 and Figure 19.

In summary, while the proposed model does not explicitly account for the influence of friction, the impact of the friction coefficient on the cutting force is relatively modest within the range of 0.2 to 0.6. This suggests that the friction coefficient plays a secondary role in determining the cutting force under the given conditions. As a result, the deviation between the calculated horizontal cutting force and the numerical simulation results remains consistent and within an acceptable tolerance across varying friction coefficients. To elaborate, when the friction coefficient is 0.2, the error range spans from −17.86% to +15.61%. For a friction coefficient of 0.4, the error range shifts slightly to −18.72% to +17.81%, indicating a marginal increase in variability. At a higher friction coefficient of 0.6, the error range expands further to −23.53% to +16.37%, reflecting a somewhat greater influence of friction at this level. Despite these variations, the errors remain within a reasonable and predictable range, underscoring the robustness of the proposed model. These findings highlight that the proposed model, even without considering friction effects, delivers reliable and consistent predictions for the horizontal cutting force within the specified friction coefficient range. This reliability is particularly valuable in practical applications, where the model can serve as a useful tool for estimating cutting forces with reasonable accuracy, even in the absence of detailed friction considerations. Furthermore, the results suggest that while friction does have an observable effect, its impact is not dominant within the tested range, reinforcing the model’s applicability for engineering purposes.

### 4.6. Effect of Initial Temperature

**Figure 20:**
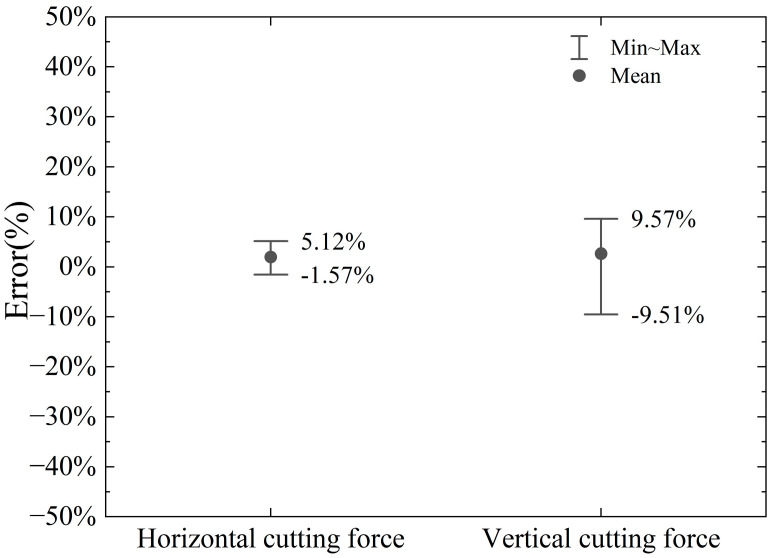
Incremental variation in the numerical model results when the initial temperature is 293.15K relative to the initial temperature of 323.15K.

**Figure 21:**
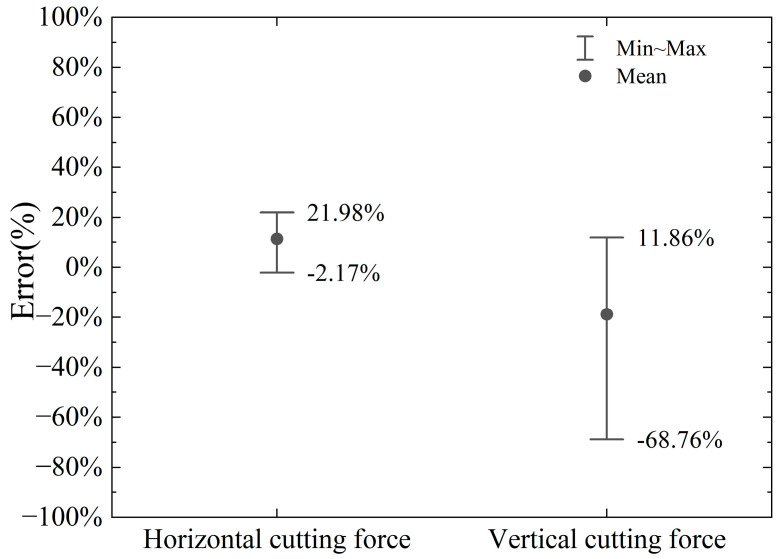
Error range of proposed model relative to numerical results for initial temperature of 293.15K.

The incremental variation in horizontal cutting force derived from numerical simulations at an initial temperature of 20 °C (293.15 K), in comparison to 50 °C (323.15 K), is systematically illustrated in Figure 20. Furthermore, Figure 21 provides a detailed representation of the error range between the cutting force predicted by the proposed model and the numerical simulation results at 20 °C (293.15 K). A comprehensive analysis of these results reveals that the initial temperature exerts a relatively limited influence on the cutting force within the range of 20 °C to 50 °C. Specifically, the cutting force at 20 °C (293.15 K) demonstrates a slight but discernible increase when compared to that at 50 °C (323.15 K). This observation underscores the fact that, within this temperature interval, the initial temperature does not induce significant changes in the cutting force dynamics. Instead, only minor variations are observed, particularly at the lower end of the temperature range. The close alignment between the proposed model’s predictions and the numerical simulation results further reinforces the model’s reliability and accuracy in capturing cutting force behavior under varying initial temperature conditions. This consistency not only validates the model’s predictive capabilities but also highlights its potential applicability in real-world scenarios where temperature fluctuations may occur. The findings suggest that, for practical purposes, the cutting force remains relatively stable across this temperature range, with only negligible adjustments required to account for the minor influence of initial temperature. This insight is particularly valuable for optimizing machining processes and ensuring precision in industrial applications where temperature control is a critical factor.

For both the numerical model and the proposed model, adiabatic conditions are assumed due to the short duration of the cutting process; this means that heat generation due to plastic deformation is not dissipated, and the temperature rise is calculated based on the plastic work. As for the proposed model, the Johnson–Cook model, it is used to calculate the maximum equivalent stress, considering the softening of the material at higher temperatures and the strain hardening and yield stress of the material. The proposed model considers the thermophysical properties in a simplified way, so the temperature gradient is not explicitly considered in the model, as the focus is on the mechanical response during the initial cutting phase.

It should also be noted that at the initial sub-zero temperature condition, different materials behave differently during the cutting process [47]. AISI 4340 steel is susceptible to phase transformations at sub-zero temperatures, forming brittle martensite and bainite, which can lead to top cracks that compromise material integrity. In contrast, AISI 304, an austenitic stainless steel, hardens through plastic deformation, forming Deformation-Hardened Martensite (DIM), which does not induce brittleness and makes it less prone to cracking at low temperatures. Similarly, AISI 1020, a low-carbon steel, retains better ductility at low temperatures and is less prone to top cracks compared to AISI 4340. Therefore, if the working conditions include sub-zero temperatures, these differences should be considered during the cutting process.

### 4.7. Effect of Circular Cross-Section Diameter

**Figure 22:**
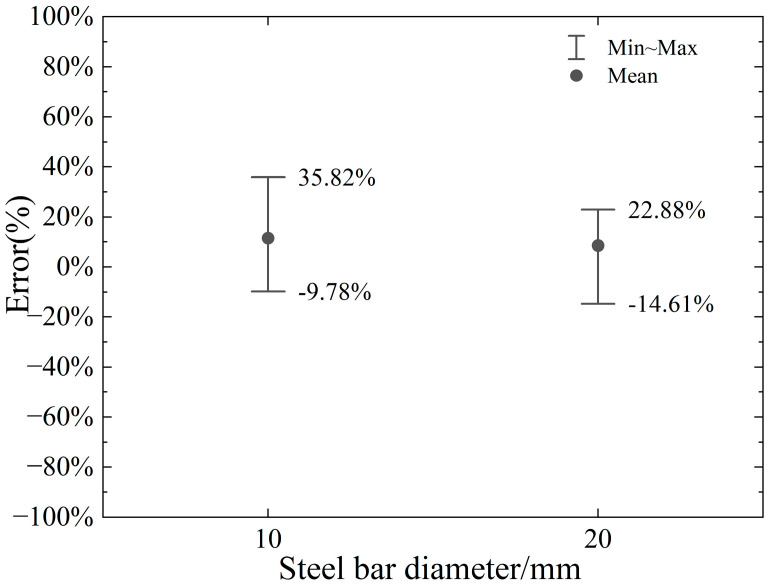
Error range in calculation of horizontal cutting force under different steel bar diameters.

**Figure 23:**
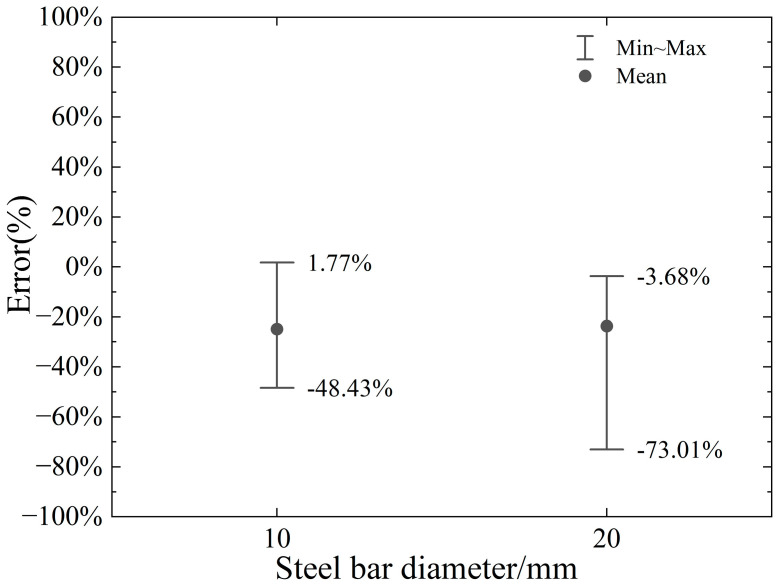
Error range in calculation of vertical cutting force under different steel bar diameters.

The error range of the proposed model relative to the numerical model in the calculation of horizontal and vertical cutting force with varying steel bar diameters is illustrated in Figure 22 and Figure 23, respectively. Analysis of the results reveals that the proposed model achieves a reasonable level of accuracy in predicting horizontal cutting force, with error ranges spanning from −9.78% to +35.82% for the 10 mm diameter and from −14.61% to +22.88% for the 20 mm diameter. These findings suggest that the model performs well in estimating horizontal cutting forces across both diameters, demonstrating its reliability for such calculations. However, the model’s performance in predicting vertical cutting force exhibits notable variability, particularly as the negative rake angle and cutting depth change. This indicates that the accuracy of vertical force calculations is highly sensitive to these specific machining parameters. While the proposed model shows promise in horizontal force prediction, the significant fluctuations in vertical force errors highlight a critical area for improvement. These insights underscore the importance of refining the model to better account for the influence of rake angle and cutting depth on vertical force, ensuring more consistent and accurate predictions across a wider range of machining conditions. Such enhancements would further strengthen the model’s applicability and precision in real-world industrial applications.

From this result, it can be concluded that extrapolating the model to greater cutting depths requires additional investigation. At larger cutting depths, the accumulation of chips in front of the cutter may become more complex, as the initial contact point between the cutter and the rebar may be slightly above the cutting edge on the rake face. Furthermore, significant deformation of the rebar may occur before the cutting edge contacts the rebar, depending on the rake angle, cutting depth, and diameter of the rebar. Alternatively, a safety coefficient may be applied to adjust the predicted cutting forces for greater depths, accounting for the simplifications inherent in the model. This coefficient can be calibrated empirically by comparing the model’s predictions at shallower depths with experimental data or more detailed numerical simulations.

## 5. Conclusions

This study introduces a model designed to assess the maximum cutting force during the initial cut of a two-dimensional circular cross-section, considering varying cutting depths of a negative rake angle cutter. The model is formulated based on insights derived from the slip plane characteristics observed in 2D numerical simulations during the early stages of cutting, integrated with slip line theory, and incorporates the effect of chip formation in front of the cutter on the slip plane. By comparing and analyzing the numerical results of two-dimensional circular cross-section cutting using the CEL model with a negative rake angle cutter, this study arrives at the following conclusions:

(1) The results confirm that cutting parameters, particularly rake angle and cutting depth, are critical in determining the required cutting forces. Optimizing these parameters can lead to more efficient cutting and reduced tool wear, especially when combined with material-specific properties.
(2) The Johnson–Cook model proves to be an effective tool for modeling cutting forces in shear cutting processes. By incorporating key material properties such as strain rate sensitivity and temperature dependence, the model provides a realistic prediction of cutting forces for different steels. This conclusion underscores the utility of the model in predicting cutting behavior across materials with varied properties.
(3) The proposed slip line model for No. 45 steel accurately describes the slip plane size and change, accounting for chip accumulation, and calculates the maximum horizontal cutting force with an error range of −11.08%~+33.85%. However, the vertical thrust force error is larger at smaller rake angles, with a maximum of −63.18%.
(4) The error in the proposed model compared to finite element simulation arises from simplifications. The model assumes uniform stress on the slip plane and ignores the cutter’s material properties, while finite element analysis accounts for dynamic stress and strain interactions across different parts of the simulation object.
(5) Chip accumulation significantly affects the slip plane size, which largely determines the horizontal cutting force at the initial cutting stage. Ignoring chip accumulation leads to increasing calculation errors as the negative rake angle increases, with errors reaching up to 57.2%.
(6) The parameter p_1_, which affects stress on the slip line, has little impact on horizontal cutting force but greatly influences vertical cutting force.
(7) Within the range of a negative rake angle of 10°~50°, the error ranges of horizontal cutting force for AISI1045 and AISI4340 are −22.56%~+18.62% and −25.69%~+12.47%. However, for AISI304, which has better toughness and temperature resistance, the model’s error is higher.
(8) A small friction coefficient (0.2~0.6) and initial temperatures of 20 °C to 50 °C have minimal impact on horizontal cutting force results. The model performs well for negative rake angles of 10° to 50° and bar diameters of 10 mm and 20 mm, with a maximum error of 35.82% for commonly used steel material. Therefore, the proposed model offers a quick preliminary estimate for horizontal cutting force but requires further refinement to reduce errors in certain conditions.
(9) The influence of initial temperature on the cutting force is relatively limited, falling within the range of 20 °C to 50 °C, and the proposed model can capture cutting force behavior under varying initial temperature conditions.
(10) The proposed model achieves quite good accuracy in predicting horizontal cutting force when the diameter of the rebar is 10 mm or 20 mm, with corresponding error ranges spanning from −9.78% to +35.82% for the 10 mm diameter and from −14.61% to +22.88% for the 20 mm diameter.

Constant thermophysical properties are assumed for computational simplicity, but this study recognizes that temperature effects could play a more significant role in cutting processes, particularly at high speeds or depths. Future work could aim to address these limitations by incorporating dynamic temperature gradients and temperature-dependent material properties, leading to more accurate predictions of cutting forces in more complex, high-temperature scenarios.

In this study, the proposed model for calculating the cutting force during the initial phase of cutting uses a simplified approach to estimate the forces acting on the cutter and the material. To improve the accuracy of the model at larger depths, future work could focus on incorporating more advanced material models, thermal–mechanical coupling, and a more detailed chip flow model.

## Figures and Tables

**Table 1:** Cutter alloy material parameters.

Parameters	Numerical Value	Unit
Density	15,700	kg/m^3^
Elastic modulus	652	GPa
Poisson’s ratio	0.22	
Thermal conductivity	75.4	75.4
Specific heat capacity	220	J/kg °C

**Table 2:** Elastic material parameters and thermodynamic parameters of No. 45 steel.

Parameters	Numerical Value	Unit
Density	7850	kg/m^3^
Elastic modulus	210	GPa
Poisson’s ratio	0.23	
Thermal conductivity	47	W/m °C
Specific heat capacity	423	J/kg °C

**Table 3:** Johnson–Cook model parameters of No. 45 steel.

Parameters	Numerical Value	Unit
A	506	MPa
B	320	MPa
C	0.064	
M	1.06	
N	0.28	
D1	0.1	
D2	0.76	
D3	1.57	
D4	0.005	
D5	0.84	
Melting temperature	1795	K
Critical temperature	293.15	K

## Data Availability

The original contributions presented in this study are included in the article. Further inquiries can be directed to the corresponding authors.

## Abbreviations

*h*	cutting depth
∆h	thickness of chip accumulation
$∆h_{ini,max}$	maximum possible thickness of chip accumulation
$∆h_{ini,min}$	minimum possible thickness of chip accumulation
$l_{G_{1}G_{2}}$	length of line segment G_1_G_2_
$l_{G_{1,i}G_{1,i}}$	length of line segment G_1,i_ G_2,i_
$l_{GV_{,ini}}$	calculated length of arc GV when ∆h= 0
$r^{\prime }$	radius of the arc slip line
*r*	radius of the circular cross-section
$S_{VG_{1}G_{2}}$	area of G_1_G_2_V region
$S_{OGG_{2}}$	area of OGG_2_ region
$S_{∆OGG_{1}}$	area of triangle OGG_1_
$S_{WMG}$	area of circular cross-section that the cutter swept
$\alpha_{n}$	absolute negative rake angle of the cutter
$\delta_{i}$	angle of rotation of tangent line
$\delta_{0}$	angles of rotation of tangent line at maximum height of accumulation
$f_{h,max}$	maximum horizontal cutting force
$f_{v,max}$	maximum vertical cutting force
${\bar{f}}_{h,sl,i}$	normalized horizontal cutting force
${\bar{f}}_{v,sl,i}$	normalized vertical cutting force
${\bar{f}}_{h,miw2,i}$	normalized cutting force obtained at he corresponding horizontal shear surface M_i_W_2_
${\bar{f}}_{h,mi}$	normalized horizontal cutting force
${\bar{f}}_{v,mi}$	normalized vertical cutting force
${\bar{\;f}}_{h,max}$	normalized maximum horizontal cutting force
${\bar{f}}_{v,max}$	normalized maximum vertical cutting force
k	flow stress on arc slip line
$\sigma_{m,si}$	average stress at point S_i_ on that slip line
$\sigma_{h,si}$	horizontal stress at point S_i_ on that slip line
$\sigma_{v,si}$	vertical stress at point S_i_ on that slip line
${\bar{\sigma }}_{h,si}$	normalized horizontal stress at point S_i_ on that slip line
${\bar{\sigma }}_{v,si}$	normalized vertical stress at point S_i_ on that slip line
*d*_1_~*d*_5_	material parameters for Johnson–Cook damage model
*c*	material specific heat capacity
*p*	average stress
*q*	Misses equivalent stress
T	material temperature
$T_{r}$	critical temperature of material
$T_{m}$	melting temperature of material
$\bar{\sigma }$	equivalent stress
${\bar{\varepsilon }}^{pl}$	cumulative equivalent plastic strain
${\dot{\bar{\varepsilon }}}^{pl}$	equivalent plastic strain rate
${\dot{\varepsilon }}_{0}$	reference strain rate
$d{\bar{\varepsilon }}^{pl}$	equivalent plastic strain increment
${\bar{\varepsilon }}_{f}^{pl}$	equivalent plastic strain when material fails
η	user-specified inelastic heat fraction
${\;\beta }_{T}$	ratio of plastic work causing temperature increase to total plastic work
ρ	material density
dT	temperature increment
$C_{0}$	ratio of the length of the slip plane $l_{s}$ to the thickness of the shear band $h_{0}$
$h_{0}$	thickness of the shear band
$l_{s}$	arc length of slip line
$l_{s,x}$	projection length of the circular slip line along the horizontal direction
n	material-related parameter, assumed to be equal to 4
$V_{s}$	shear velocity on the slip plane
$V_{h}$	horizontal cutting speed
$\bar{x}$	normalized distance relative to $h_{0}$
${\dot{\gamma }}_{s}$	shear strain rate on the slip plane
$\dot{\gamma }$	shear strain rate at distance x from the shear plane in the shear band
λ	parameter used in shear band model, assumed to be equal to 0.5
$p_{1}$	parameter facilitating analysis of the effect of changes in pressure on the slip line on cutting force

## Appendix A

In this study, numerical simulation methodology is used, for which the coupled Eulerian–Lagrangian (CEL) method in Abaqus is employed. A detailed explanation of the assumptions, mesh settings, governing equations, and boundary conditions employed in the coupled Eulerian–Lagrangian (CEL) method used for simulating the orthogonal cutting process with a negative rake angle cutter in Abaqus is given in the following section.

### Appendix A.1. Assumptions

This section outlines the key assumptions made in the modeling and simulation of the 2D cutting process. The assumptions span geometry, material behavior, and the cutting process itself to ensure a consistent and realistic representation of the system in the numerical model. These assumptions are critical for simplifying the complexity of the cutting process while capturing its essential features, including the effects of material deformation, thermal interactions, and tool characteristics. The following assumptions are made:

- Geometry Modeling:

For modeling the 2D cutting process, as the CEL method can only be used in 3D models in Abaqus, the model thickness is set to 0.001 m, and the displacement along thickness direction is assumed to be 0.

- Material Behavior:

The material of the steel bars is modeled using the Johnson–Cook plasticity model and Johnson–Cook damage model. Due to the material model used, the material will undergo plastic deformation, thermal softening, and damage accumulation during the simulation of the cutting process.

Diabatic conditions are assumed in the numerical model due to the short duration of the cutting process, and the thermal softening is reflected through the adopted Johnson–Cook model.

- Cutting Process:

The cutting process is assumed to be adiabatic. The cutting tool is a negative rake angle cutter, which is modeled as an elastic body.

### Appendix A.2. Mesh Settings

The mesh resolution and element types are chosen to balance computational efficiency with the need for accurate representation of the cutting process, especially in the regions where the most significant interactions occur. The following mesh configurations are applied:

- Workpiece (Steel Bar):

The workpiece is discretized using Eulerian mesh elements, with a mesh size of 0.5 mm in the main cutting region. Eight-node, thermally coupled, reduced integration Eulerian elements (EC3D8RT) are used to model the workpiece, allowing for the accurate capture of material deformation and thermal effects during the cutting process.

- Cutter (Negative Rake Angle Cutter):

The cutter is modeled with Lagrangian solid elements to maintain its geometry throughout the simulation. The mesh size at the main contact region of the cutter is 1 mm, which is also the smallest mesh size for the cutter. Eight-node, reduced integration solid elements (C3D8R) are employed to model the cutter, ensuring precise interaction with the workpiece during the cutting process.

### Appendix A.3. Governing Equations

- CEL Governing Equations:

The coupled Eulerian–Lagrangian (CEL) method was formulated with the aim of combining the advantages of the Lagrangian and Eulerian formulations and allows Eulerian and Lagrangian bodies within the same model to interact. The conservative forms of Eulerian equations [48,49], i.e., the mass, momentum, and energy equations, are as follows:

(A1) $$\frac{\partial \rho }{\partial t}+\nabla \cdot \left(\rho v\right)=0$$

(A2) $$\frac{\partial \rho v}{\partial t}+\nabla \cdot \left(\rho v⊗v\right)=\nabla \cdot \sigma +\rho b$$

(A3) $$\frac{\partial e}{\partial t}+\nabla \cdot \left(ev\right)=\sigma :D$$

where *b* is the vector of body forces, *D* is the velocity strain, *e* is the internal energy, *v* is the material velocity, ρ is the density, and σ is the Cauchy stress.

The Eulerian governing equations have a general form:

(A4) $$\frac{\partial \varphi }{\partial t}+\nabla \cdot \Phi =S$$

where Φ is the flux function and S is the source term.

Operator splitting divides the general form into two equations, as follows:

(A5) $$\frac{\partial \varphi }{\partial t}=S$$

(A6) $$\frac{\partial \varphi }{\partial t}+\nabla \cdot \Phi =0$$

Equation (A5) contains the source term that represents the Lagrangian step, and Equation (A6) contains the convective term that represents the Eulerian step. These two equations are then solved sequentially.

- Johnson–Cook Model:

The Johnson–Cook model is a plasticity damage model that is based on Mises plasticity, in accordance with strain hardening, strain rate hardening, thermal softening, and damage accumulation.

(A7) $$\bar{\sigma }=\left(1-D\right)\left[A+B{\left({\bar{\varepsilon }}^{pl}\right)}^{n}\right]\left[1+C\ln\frac{{\dot{\bar{\varepsilon }}}^{pl}}{{\dot{\varepsilon }}_{0}}\right]\left[1-{\left(\frac{T-T_{r}}{T_{m}-T_{r}}\right)}^{m}\right]$$

(A8) $${\bar{\varepsilon }}_{f}^{pl}=\left[d_{1}+d_{2}exp\left(-d_{3}\eta \right)\right]\left[1+d_{4}\ln\left(\frac{{\dot{\bar{\varepsilon }}}^{pl}}{{\dot{\varepsilon }}_{0}}\right)\right]\left[1+d_{5}\frac{T-T_{r}}{T_{m}-T_{r}}\right]$$

(A9) $$D=\sum \frac{d{\bar{\varepsilon }}^{pl}}{{\bar{\varepsilon }}_{f}^{pl}}$$

where A, B, C, m, and n are the material parameters of the Johnson–Cook plasticity model, *d*_1_~*d*_5_ are the material parameters for the Johnson–Cook damage model, D is the damage value, T is the material temperature, $T_{r}$ is the critical temperature, $T_{m}$ is the melting temperature of the material, ${\bar{\varepsilon }}^{pl}$ is the cumulative equivalent plastic strain, ${\dot{\bar{\varepsilon }}}^{pl}$ is the equivalent plastic strain rate, ${\dot{\varepsilon }}_{0}$ is the reference strain rate, η = −*p*/*q*, where *p* is the average stress, *q* is the Misses equivalent stress, and $\bar{\;\sigma }$ is the equivalent stress.

### Appendix A.4. Conservation of Energy (Thermal Effects):

The increase in temperature is calculated directly at the material integration points based on the increase in adiabatic thermal energy caused by inelastic deformation. Temperature is not a degree of freedom in the analysis, and there is conduction of heat. The conservation of energy is taken into consideration, as follows:

(A10) $$\rho c\dot{T}=\beta_{T}\sigma :{\dot{\varepsilon }}^{pl}$$

where *c* is the specific heat, $\dot{T}$ is the rate of the temperature increase, ${\dot{\varepsilon }}^{pl}$ is the rate of plastic straining, $\beta_{T}$ is the user-specified inelastic heat fraction, *ρ* is the material density, and σ is the stress.

### Appendix A.5. Boundary Conditions

- Workpiece (Steel Bar):

For the workpiece, which is a steel bar, the CEL method can only be applied to 3D models in Abaqus. Therefore, for the surface of the workpiece that is perpendicular to the thickness direction, the displacement along this direction is constrained. For surfaces that are not perpendicular to the thickness direction, the normal displacement is constrained at the surface boundary below the cutting depth (h + 0.4 mm) of the workpiece or steel bar.

- Cutter (Negative Rake Angle Cutter):

The cutter is treated as an elastic body in the simulation. It moves at a constant velocity along the cutting path. As for the workpiece (steel bar), the displacement of the surface that is vertical to the thickness direction is constrained in the direction of thickness. The cutter’s motion is defined in terms of its velocity vector along the cutting direction.

### Appendix A.6. Computational Details

The numerical simulation is performed using Abaqus software, with the CEL method implemented to model the interaction between the cutter and the workpiece. The following details apply to the computational setup:

○ Explicit time integration is used.
○ Abaqus/Explicit, an explicit dynamic finite element solver, is used.
○ The analysis step of the type “Dynamic, Explicit” is used in the simulation.
○ The time step is adjusted automatically (an option in Abaqus), and the option for “include adiabatic heating effects” is selected.
○ Abaqus/Explicit solver is used for the simulation.

## References

[1] Xu Q., Zhu H., Ma X., Ma Z., Li X., Tang Z., Zhuo K. (2015). A Case History of Shield Tunnel Crossing through Group Pile Foundation of a Road Bridge with Pile Underpinning Technologies in Shanghai. Tunn. Undergr. Space Technol..

[2] Chen H., Yuan D., Wang F., Wang M. (2016). Study on Shield Cutting Parameters When Cutting Big Diameter Piles. China Civ. Eng. J..

[3] Wang G., Qiao S., Wang G., Jiang Q., Singh J. (2023). Determination and Application of Optimum Abrasive Mass Flow Rate of Abrasive Waterjet. KSCE J. Civ. Eng..

[4] Wang X., Yuan D. (2023). Research on the Interaction between the Pile and Shield Machine in the Process of Cutting a Reinforced Concrete Pile Foundation. Appl. Sci..

[5] Wang G., Qiao S., Li G., Singh J. (2023). Direct Shield Cutting of Large-Diameter Reinforced Concrete Group Piles: Case Study on Shenyang Metro Construction. Case Stud. Constr. Mater..

[6] Wu D. (2021). Analysis of Influence of Shield Tunnel Cutting Building Pile Foundation Groups Under Complicated Geological Conditions. Urban Mass Transit..

[7] Xin D., Huang X., Yan H., Wei L. (2021). Analysis on Cutterhead Load in Shield Cutting Concrete Bridge Pile. Chin. J. Undergr. Space Eng..

[8] Peng F., Ma S., Li M., Fu K. (2022). Stress Performance Evaluation of Shield Machine Cutter Head during Cutting Piles under Masonry Structures. Adv. Civ. Eng..

[9] Zhang C., Ma S., Guo Y., Li M., Fu K. (2022). Shield Cutting Pile-Group Implementation Effects on the Superstructure. Adv. Civ. Eng..

[10] Bai C., Liu K., Zhao T., Liu J. (2022). Study on Spray Characteristics and Breakup Mechanism of an SCR Injector. Appl. Sci..

[11] Tang R., Lin B.H., Liang P. (2019). Study on the Safety of Shield Passing through the Residential Building and Directly Cutting Pile Foundation. Chin. J. Undergr. Space Eng..

[12] Zhang C., Zhao Y., Zhang Z., Zhu B. (2021). Case Study of Underground Shield Tunnels in Interchange Piles Foundation Underpinning Construction. Appl. Sci..

[13] Du C., Zhang J., Tang O. (2019). Key Technologies of Shield Direct Cutting Pile Foundation. Tunn. Constr..

[14] Wang Y., Li J., Liao S. (2017). Numerical Simulation and Measured Data Analysis of Pile Group Cutting by Shield: A Case Study of Running Tunnel on Line No. 9 of Shenzhen Metro. Tunn. Constr..

[15] Wang Z., Wu S.W., Yao W.J., Zhang K.W., Li Q., Xu S.F. (2020). Grinding Pile Technology of Shield Tunnels Crosssing Pile Foundation of Existing Bridges. Chin. J. Geotech. Eng..

[16] Wang F., Yuan D.J., Cai R., Mu Y.J., Wang M.S. (2013). Field Test Study on Cutting Obstacle Piles Directly by Shield Cutters. Appl. Mech. Mater..

[17] Liu B., Li T., Han Y., Li D., He L., Fu C., Zhang G. (2022). DEM-Continuum Mechanics Coupling Simulation of Cutting Reinforced Concrete Pile by Shield Machine. Comput. Geotech..

[18] Wang Y., Wang X., Xiong Y., Yang Z., Zhang J. (2022). Full-Scale Laboratory Test of Cutting Large-Diameter Piles Directly by Shield Cutterhead. Adv. Civ. Eng..

[19] Yuan D.J., Wang F., Dong C.W., Han B., Wang M.S. (2016). Study on New-Style Cutter for Shield Cutting Large-Diameter Reinforced Concrete Pile. China J. Highw. Transp..

[20] Wang F. (2014). Study on Shield Cutting Large Diameter Reinforced Concrete Piles Directly.

[21] Su W.L., Li X.G., Xu Y., Jin D.L. (2020). Numerical Simulation of Shield Tool Cutting Concrete Based on HJC Model. J. Zhejiang Univ. Eng. Sci..

[22] Xu P., Zuo S. (2021). Study on the JH-2 Model Parameters for Metro Shield Cutting Reinforced Concrete Pile. Geotech. Geol. Eng..

[23] Li H. (2020). Feasibility Study on Direct Cutting of Reinforced Concrete Pile Foundation with Φ25 Mm Main Reinforced Bar by Shield. Tunn. Constr..

[24] Wang F., Yuan D., Dong C., Han B., Nan H., Wang M. (2013). Test Study of Shield Cutting Large-Diameter Reinforced Concrete Piles Directly. Chin. J. Rock Mech. Eng..

[25] Wang F., Yuan D., Dong C., Han B., Nan H., Wang M. (2013). Study on Cutter Configuration for Directly Shield Cutting of Large-Diameter Piles. China Civ. Eng. J..

[26] Xu H., Chen K., Sun Z. (2020). Laboratory Test of Reinforced Concrete Pile Foundation Cutting by Shield Cutterhead. Tunn. Constr..

[27] Huang X., Liu Q., Chen L., Pan Y., Liu B., Kang Y., Liu X. (2018). Cutting Force Measurement and Analyses of Shell Cutters on a Mixshield Tunnelling Machine. Tunn. Undergr. Space Technol..

[28] Guan X., Liu Z., Xu H., Liu Y., Ling X., Ding H., Ren S., Lu R., Yu K., Miao J. (2023). Mechanical Properties and Influencing Factors of Shield Cutting Existing Station Supporting Piles. Sustainability.

[29] Fu D. (2014). Model Test on Concrete Cutting Directly by Shield and Pile Foundation Cutting Technology. Tunn. Constr..

[30] Ye S., Wang J., Fan H., Zhang Z. (2021). Probabilistic Model for Truth Discovery with Mean and Median Check Framework. Knowl.-Based Syst..

[31] Chen Z.S., Yang L.L., Chin K.S., Yang Y., Pedrycz W., Chang J.P., Martínez L., Skibniewski M.J. (2021). Sustainable Building Material Selection: An Integrated Multi-Criteria Large Group Decision Making Framework. Appl. Soft Comput..

[32] Yin Q. (2022). Design and Application of Smart City Internet of Things Service Platform Based on Fuzzy Clustering Algorithm. Mob. Inf. Syst..

[33] Wang Q., Li W., Wang D. Simulation Research on Cutting of Shield Machine Cutter Tool Based on Anisotropic Composite Materials. Proceedings of the International Conference on Advances in Construction Machinery and Vehicle Engineering.

[34] Altan E., Emiroğlu U. (2022). New Slip-Line Field Model Based on Dead Metal Zone and Material Flow in Negative Rake Orthogonal Cutting. J. Braz. Soc. Mech. Sci. Eng..

[35] Hu C., Zhuang K., Weng J., Zhang X. (2019). Thermal-Mechanical Model for Cutting with Negative Rake Angle Based on a Modified Slip-Line Field Approach. Int. J. Mech. Sci..

[36] Ozturk S., Altan E. (2012). Slip-Line Metal Cutting Model with Negative Rake Angle. J. Braz. Soc. Mech. Sci. Eng..

[37] Jin X., Altintas Y. (2011). Slip-Line Field Model of Micro-Cutting Process with Round Tool Edge Effect. J. Mater. Process. Technol..

[38] Merchant M.E. (1945). Mechanics of the Metal Cutting Process. I. Orthogonal Cutting and a Type 2 Chip. J. Appl. Phys..

[39] Zhou J., Ren J. (2020). Predicting Cutting Force with Unequal Division Parallel-Sided Shear Zone Model for Orthogonal Cutting. Int. J. Adv. Manuf. Technol..

[40] Zhou J., Ren J., Feng Y., Tian W., Shi K. (2017). A Modified Parallel-Sided Shear Zone Model for Determining Material Constitutive Law. Int. J. Adv. Manuf. Technol..

[41] Lalwani D.I., Mehta N.K., Jain P.K. (2009). Extension of Oxley’s Predictive Machining Theory for Johnson and Cook Flow Stress Model. J. Mater. Process. Technol..

[42] Yang G. (2013). Introduction to Elastic-Plastic Mechanics.

[43] Shi B., Attia H., Tounsi N. (2010). Identification of Material Constitutive Laws for Machining-Part I: An Analytical Model Describing the Stress, Strain, Strain Rate, and Temperature Fields in the Primary Shear Zone in Orthogonal Metal Cutting. J. Manuf. Sci. Eng..

[44] Zhu B., Xiong L., Xu M. (2022). Double-Edged Cutting Simulation with a New Combined Constitutive Model for AISI 1045 Steel. J. Mater. Process. Technol..

[45] Sulaiman S., Roshan A., Ariffin M.K.A. (2013). Finite Element Modelling of the Effect of Tool Rake Angle on Tool Temperature and Cutting Force during High Speed Machining of AISI 4340 Steel. IOP Conf. Ser.: Mater. Sci. Eng..

[46] Zou Z., He L., Zhou T., Wang M., Tian P., Zhou X. (2022). Research on Microhardness Prediction of 304 Stainless Steel Turning Based on Dislocation Density. J. Manuf. Process..

[47] Valtonen K., Ratia V., Ramakrishnan K.R., Apostol M., Terva J., Kuokkala V.T. (2019). Impact Wear and Mechanical Behavior of Steels at Subzero Temperatures. Tribol. Int..

[48] Benson D.J., Okazawa S. (2004). Contact in a multi-material Eulerian finite element formulation. Comput. Methods Appl. Mech. Eng..

[49] Skrzat A. (2012). Application of coupled Eulerian-Lagrangian approach in metal forming simulations. Zeszyty Naukowe Politechniki Rzeszowskiej. Mechanika.

